# Risk of lung cancer associated with residential radon exposure in south-west England: a case-control study.

**DOI:** 10.1038/bjc.1998.506

**Published:** 1998-08

**Authors:** S. Darby, E. Whitley, P. Silcocks, B. Thakrar, M. Green, P. Lomas, J. Miles, G. Reeves, T. Fearn, R. Doll

**Affiliations:** ICRF Cancer Epidemiology Unit, University of Oxford, Radcliffe Infirmary, UK.

## Abstract

Studies of underground miners occupationally exposed to radon have consistently demonstrated an increased risk of lung cancer in both smokers and non-smokers. Radon exposure also occurs elsewhere, especially in houses, and estimates based on the findings for miners suggest that residential radon is responsible for about one in 20 lung cancers in the UK, most being caused in combination with smoking. These calculations depend, however, on several assumptions and more direct evidence on the magnitude of the risk is needed. To obtain such evidence, a case-control study was carried out in south-west England in which 982 subjects with lung cancer and 3185 control subjects were interviewed. In addition, radon concentrations were measured at the addresses at which subjects had lived during the 30-year period ending 5 years before the interview. Lung cancer risk was examined in relation to residential radon concentration after taking into account the length of time that subjects had lived at each address and adjusting for age, sex, smoking status, county of residence and social class. The relative risk of lung cancer increased by 0.08 (95% CI -0.03, 0.20) per 100 Bq m(-3) increase in the observed time-weighted residential radon concentration. When the analysis was restricted to the 484 subjects with lung cancer and the 1637 control subjects with radon measurements available for the entire 30-year period of interest, the corresponding increase was somewhat higher at 0.14 per 100 Bq m(-3) (95% CI 0.01, 0.29), although the difference between this group and the remaining subjects was not statistically significant. When the analysis was repeated taking into account uncertainties in the assessment of radon exposure, the estimated increases in relative risk per 100 Bq m(-3) were larger, at 0.12 (95% CI -0.05, 0.33) when all subjects were included and 0.24 (95% CI -0.01, 0.56) when limited to subjects with radon measurements available for all 30 years. These results are consistent with those from studies of residential radon carried out in other countries in which data on individual subjects have been collected. The combined evidence suggests that the risk of lung cancer associated with residential radon exposure is about the size that has been postulated on the basis of the studies of miners exposed to radon.


British Journal of Cancer (1998) 78(3), 394–408
© 1998 Cancer Research Campaign

### Risk of lung cancer associated with residential radon exposure in south-west England: a case–control study

S Darby[1], E Whitley[1,2], P Silcocks[1,3], B Thakrar[1,4], M Green[5], P Lomas[5], J Miles[5], G Reeves[1], T Fearn[6] and R Doll[7]

[1]ICRF Cancer Epidemiology Unit, University of Oxford, Gibson Building, Radcliffe Infirmary, Oxford OX2 6HE; [2]Department of Social Medicine, University of Bristol, Canynge Hall, Whiteladies Road, Bristol BS8 2PR; [3]Trent Institute for Health Services Research, Queens Medical Centre, Nottingham NG7 2UH; [4]Glaxo Wellcome, Medical Data Sciences, Greenford Road, Greenford, Middlesex UB6 0HE; [5]National Radiological Protection Board, Chilton, Didcot, Oxon OX11 0RQ; [6]Department of Statistical Science, University College London, Gower Street, London WC1E 6BT; [7]Clinical Trial Service Unit, University of Oxford, Harkness Building, Radcliffe Infirmary, Oxford OX2 6HE, UK

**Summary** Studies of underground miners occupationally exposed to radon have consistently demonstrated an increased risk of lung cancer in both smokers and non-smokers. Radon exposure also occurs elsewhere, especially in houses, and estimates based on the findings for miners suggest that residential radon is responsible for about one in 20 lung cancers in the UK, most being caused in combination with smoking. These calculations depend, however, on several assumptions and more direct evidence on the magnitude of the risk is needed. To obtain such evidence, a case–control study was carried out in south-west England in which 982 subjects with lung cancer and 3185 control subjects were interviewed. In addition, radon concentrations were measured at the addresses at which subjects had lived during the 30-year period ending 5 years before the interview. Lung cancer risk was examined in relation to residential radon concentration after taking into account the length of time that subjects had lived at each address and adjusting for age, sex, smoking status, county of residence and social class. The relative risk of lung cancer increased by 0.08 (95% CI –0.03, 0.20) per 100 Bq $m^{-3}$ increase in the observed time-weighted residential radon concentration. When the analysis was restricted to the 484 subjects with lung cancer and the 1637 control subjects with radon measurements available for the entire 30-year period of interest, the corresponding increase was somewhat higher at 0.14 per 100 Bq $m^{-3}$ (95% CI 0.01, 0.29), although the difference between this group and the remaining subjects was not statistically significant. When the analysis was repeated taking into account uncertainties in the assessment of radon exposure, the estimated increases in relative risk per 100 Bq $m^{-3}$ were larger, at 0.12 (95% CI –0.05, 0.33) when all subjects were included and 0.24 (95% CI –0.01, 0.56) when limited to subjects with radon measurements available for all 30 years. These results are consistent with those from studies of residential radon carried out in other countries in which data on individual subjects have been collected. The combined evidence suggests that the risk of lung cancer associated with residential radon exposure is about the size that has been postulated on the basis of the studies of miners exposed to radon.

**Keywords:** case–control study; lung cancer; radon; measurement error; risk analysis

Studies of mortality patterns among underground miners exposed occupationally to the natural radioactive gas radon-222 and its decay products have consistently demonstrated an increased risk of lung cancer in both smokers and non-smokers (National Research Council, 1998). These observations have been confirmed by experimental studies in rats (Cross, 1994), and radon has been classified as a human carcinogen by the International Agency for Research on Cancer (IARC, 1988). Radon is not, however, confined to underground mines, and surveys have suggested that radon accounts for approximately half the average annual effective dose of ionizing radiation received by the UK population, amounting to about 1.2 mSv per year out of a total of 2.5 mSv per person (Clarke and Southwood, 1989). Most radon exposure occurs indoors, predominantly in the home, and it has been estimated that, in dwellings in the UK, the average concentration of radon gas is around 20 Bq $m^{-3}$ (Wrixon et al, 1988). There is, however, a wide range of values across the country, with the highest levels occurring, in general, in Devon and Cornwall in south-west England.

Based on estimates of the risk of lung cancer derived from studies of underground miners, it has been suggested that residential radon is responsible for approximately one in 20 lung cancers occurring in the UK, most being caused in combination with smoking (NRPB, 1990). Calculations such as these depend, however, on several assumptions and are subject to considerable uncertainty. One of these assumptions concerns the extent to which estimates of the lung cancer risk derived from studies of underground miners are applicable to residential radon. We have therefore sought to provide direct evidence by means of a case–control study of radon and lung cancer in long-term residents of Devon and Cornwall.

### METHODS

### Relevant period of exposure

In conducting this study, it has been assumed that the period of exposure to residential radon that is relevant to the risk of lung cancer at a particular point in time is the 30-year period ending 5 years previously. This period has been chosen based on the studies of underground miners in which exposure within the previous 5 years and exposure more than 35 years previously were found to

*Received 10 February 1998*
*Revised 12 March 1998*
*Accepted 17 March 1998*

*Correspondence to:* SC Darby, ICRF Cancer Epidemiology Unit, Gibson Building, Radcliffe Infirmary, Oxford OX2 6HE, UK

have little or no effect on the risk of the disease (Tomasek et al. 1994; Lubin et al. 1995*a*).

### Study subjects

At each of the five centres in Devon and Cornwall where investigation and treatment of lung cancer is carried out, all subjects aged less than 75 years who were referred with a suspected diagnosis of lung cancer during a 4-year period were identified each week by local research assistants. The centres and periods involved were: Plymouth July 1988–June 1992, Barnstaple May 1989–April 1993, Truro May 1989–April 1993, Torquay June 1989–May 1993 and Exeter July 1989–June 1993. Subjects were eligible for the study if they were current residents of the counties of Devon or Cornwall and had lived in either county for at least 20 years during the 30-year period ending 5 years previously. Only subjects who were ethnically white were included in the study as very few residents of Devon or Cornwall are from other ethnic groups, making the identification of control subjects of similar age, sex and ethnic group extremely difficult. In all, 2959 subjects with suspected lung cancer were identified (Table 1). Of these, 1175 (39.7%) did not satisfy the residence requirements. A total of 1412 (47.7%) did satisfy the requirements and were interviewed by a local research assistant using a structured questionnaire. The remaining subjects were not interviewed for a variety of reasons: the responsible medical staff withheld permission in 260 cases (8.8%), usually because the subject was very ill; the research assistants thought a further nine subjects (0.3%) were too ill to question; 31 (1.0%) died before they could be questioned; 68 (2.3%) did not wish to participate; and four (0.1%) were non-white and therefore ineligible.

For each subject with suspected lung cancer who was interviewed, a control was sought from hospital patients of the same sex, born within 5 years of the case, who satisfied the study residence requirements, and whose current hospital admission was for a disease not known to be strongly associated with smoking. Patients whose current hospital admission was for a disease closely associated with smoking (see Table 2 for list of diseases) were excluded so that smokers would not be over-represented in the hospital control group compared with the population from which they were drawn. As referral patterns differed between patients with suspected lung cancer and other diseases, patients at each centre were also matched on two or three broad residential areas, based on county districts appropriate for the relevant centre: namely (1) Plymouth vs elsewhere, (2) North Devon and Torridge vs elsewhere, (3) West Cornwall (Kerrier and Penwith) vs mid-Cornwall (Carrick) vs elsewhere, (4) Torbay vs elsewhere and (5) Exeter vs elsewhere. To select hospital controls, each research assistant had a list of hospital wards. Each week, as the starting point, one ward was selected randomly, with probability proportional to the number of beds. Patients in that ward were then considered systematically, to see if any fulfilled the matching criteria, and then patients on the next ward in the list were considered, and so on. A total of 2401 subjects were approached as hospital controls, of whom 1418 (59.1%) were interviewed; 881 (36.7%) did not satisfy the residence requirements; permission to interview was withheld by the medical staff for 65 (2.7%); 35 (1.5%) did not wish to participate; and two (0.1%) were non-white and therefore ineligible.

Some time after the interview, the hospital case notes of the subjects with suspected lung cancer were reviewed (see Table 2). For 982 of the 1412 subjects, the final diagnosis was primary cancer of the trachea, bronchus or lung [International Classification of Diseases, 9th revision, code 162 (World Health Organization, 1975), but excluding carcinoids]. The original pathological slides for these patients were reviewed blind by one of us who had histopathological training (PS) and coded according to the International Classification of Diseases for Oncology (ICD-O) (World Health Organization, 1976). The ICD-O codes were then aggregated into groups. Confirmation of the diagnosis was available by histology for 696 (70.9%) of the subjects whose final diagnosis was lung cancer, and by cytology for a further 140 (14.3%); no microscopic evidence was available for 146 (14.9%). In this last group, the clinical outcomes and the proportion who were life-long non-smokers provide evidence that the majority had been correctly identified as having lung cancer. By the end of the investigation, 73% of the subjects without microscopic confirmation had died and 91% of these patients were certified as having died of lung cancer against 76% and 97%, respectively (indirectly standardized for age), of those with microscopic confirmation. The proportions of life-long non-smokers in the two groups were 0.0% of those without and 0.5% of those with microscopic confirmation in men and 8.3% and 7.1%, respectively, in women.

Of the remaining subjects originally suspected to have lung cancer, the final diagnosis was a smoking related disease (see Table 2) in 113, and these were excluded from the study, while the other 317 were transferred to the hospital control group. The hospital case notes of subjects selected as hospital controls were also reviewed. For 36 patients, the final diagnosis was a smoking related disease and they were excluded from the study. The final diagnoses of the remaining 1382 patients and the 317 transferred from the suspected lung cancer group are listed in broad categories in Table 2.

In addition to the hospital controls, a further population-based group of controls was selected, frequency-matched to the subjects with suspected lung cancer by age, sex and county of residence. In Cornwall, these controls were randomly selected from the lists of the Family Health Services Authority (FHSA) (formerly Family Practitioner Committee), and permission for interview was sought from each patient's general practitioner before contact was made. Population controls in Devon were initially selected in the same way but, during the course of the study, permission to use FHSA lists was withdrawn, and the remaining controls were randomly selected using electoral rolls. A total of 5223 individuals were selected as population controls, including 2444 from FHSA lists and 2779 from electoral rolls (Table 1). Of these, 1486 (28.5%) were interviewed; 1119 (21.4%) did not satisfy the residence requirements; 304 (5.8%) did not wish to take part; 160 (3.1%) were thought by the general practitioner to be unsuitable to approach, usually because the subject or a family member was unwell; 43 (0.8%) were judged too ill by the research assistant; 199 (3.8%) were found to have died; 291 (5.6%) had moved to an unknown address; and the remaining 1621 (31.0%) were ineligible, either because they had moved outside the study area, were outside the age range, were in an age/sex band for which sufficient subjects (i.e. as many as the final number of subjects with suspected lung cancer) had already been interviewed, were already in the study or were of non-white ethnic group.

When the hospital and population controls were compared with respect to smoking status (see section entitled Information on other factors for description), length of residence in Devon or Cornwall, and the number of addresses in the 30-year period of

© Cancer Research Campaign 1998

**Table 1** Outcome of approach to subjects with suspected lung cancer and to control subjects

| Outcome | Subjects with suspected lung cancer Number (%) | Hospital controls Number (%) | Population controls | |
|---|---|---|---|---|
| | | | Family Health Services Authority Number (%) | Electoral roll Number (%) |
| Interviewed | 1412 (47.7) | 1418 (59.1) | 1059 (43.3) | 427 (15.4) |
| Residence in study area too short | 1175 (39.7) | 881 (36.7) | 774 (31.7) | 345 (12.4) |
| Medical staff refused[a] | 260 (8.8) | 65 (2.7) | 160 (6.5) | – – |
| Too ill[b] | 9 (0.3) | – – | 18 (0.7) | 25 (0.9) |
| Died | 31 (1.0) | – – | 102 (4.2) | 97 (3.5) |
| Moved to unknown address | – – | – – | 88 (3.6) | 203 (7.3) |
| Subject refused | 68 (2.3) | 35 (1.5) | 150 (6.1) | 154 (5.5) |
| Ineligible | 4[c] (0.1) | 2[c] (0.1) | 93[d] (3.8) | 1528[e] (55.0) |
| Total number approached | 2959 (100.0) | 2401 (100.0) | 2444 (100.0) | 2779 (100.0) |

[a]Usually because subject or a family member was very ill. [b]As judged by local research assistants. [c]Non-white ethnic group. [d]Moved outside study area (50), subsequently found to be outside age range (31), already in study (10), non-white ethnic group (2). [e]Age/sex group already full (897), outside age range (622), already in study (9).

**Table 2** Final diagnosis of subjects selected with suspected lung cancer and subjects selected as hospital controls

| Final diagnosis | Subjects selected with suspected lung cancer | Subjects selected as hospital controls |
|---|---|---|
| Lung cancer | 982 | 0 |
| Histological confirmation | 696 | – |
| Cytological confirmation only | 140 | – |
| No microscopic evidence | 146 | – |
| No lung cancer, but disease associated with smoking[a] | 113 | 36 |
| Other diseases | 317[b] | 1382 |
| Cancer of large bowel | 5 | 71 |
| Other neoplasms | 65 | 121 |
| Diseases of central nervous system and sense organs | 4 | 93 |
| Other respiratory disease | 164 | 25 |
| Hernia | 2 | 91 |
| Gall bladder disease | 0 | 63 |
| Other digestive system | 2 | 124 |
| Prostatic hypertrophy | 0 | 118 |
| Other genitourinary disease | 0 | 109 |
| Osteoarthritis | 0 | 106 |
| Other musculoskeletal disease | 3 | 96 |
| Other defined disease | 34 | 119 |
| Ill-defined disease | 37 | 54 |
| Injury and poisoning | 1 | 192 |
| Total number of subjects | 1412 | 1418 |

[a]Coronary heart disease, chronic bronchitis, peripheral vascular disease, aortic aneurysm, stroke, peptic ulcer, cirrhosis of liver, tuberculosis, road traffic accidents or burns attributed to alcohol consumption of subject, and cancers of lip, mouth, pharynx, larynx, oesophagus, pancreas, kidney, bladder, cervix and unknown primary site. These subjects were excluded from the study. [b]These subjects were transferred to the hospital control group.

interest, patterns in the two groups were very similar for each sex (Tables 3 and 4). The two control groups were therefore combined for examination of radon-related risk. The final number of subjects included in the analysis was 4167, comprising 982 subjects with lung cancer and 3185 controls.

### Information on residential radon concentrations

For all subjects who were interviewed, full residential histories covering the previous 35 years were obtained. For each dwelling at which the subject had been a resident for more than a year, information was collected on the precise address, the period it was occupied by the subject and the following housing characteristics, which were noted by Gunby et al (1993) as having the greatest bearing on residential radon levels in the UK: type of building, floor levels of living area and bedroom, and presence of double-glazing in living area and bedroom. Attempts were made to measure the radon concentration in every address in Devon or Cornwall at which the subjects had lived during the 30-year period of interest. Two detectors were installed for a period of 6 months,

 © Cancer Research Campaign 1998

**Table 3** Distribution of subjects by smoking status

| Smoking status | Lung cancer | | | | Hospital controls | | | | Population controls | | | |
|---|---|---|---|---|---|---|---|---|---|---|---|---|
| | Male | | Female | | Male | | Female | | Male | | Female | |
| | No. | (%) | No. | (%) | No. | (%) | No. | (%) | No. | (%) | No. | (%) |
| Life-long non-smoker[a] | 3 | (0.4) | 23 | (7.3) | 189 | (16.9) | 274 | (47.2) | 195 | (19.7) | 255 | (51.3) |
| Current cigarette (<15 per day) | 128 | (19.2) | 71 | (22.5) | 113 | (10.1) | 58 | (10.0) | 110 | (11.1) | 46 | (9.3) |
| Current cigarette (15–24 per day) | 126 | (18.9) | 86 | (27.3) | 98 | (8.8) | 54 | (9.3) | 71 | (7.2) | 38 | (7.7) |
| Current cigarette (25+ per day) | 68 | (10.2) | 38 | (12.1) | 40 | (3.6) | 15 | (2.6) | 21 | (2.1) | 6 | (1.2) |
| Ex-smoker (<10 years)[b] | 146 | (21.9) | 68 | (21.6) | 177 | (15.8) | 57 | (9.8) | 162 | (16.4) | 36 | (7.2) |
| Ex-smoker (10+ years)[b] | 139 | (20.8) | 26 | (8.3) | 412 | (36.8) | 117 | (20.2) | 355 | (35.9) | 107 | (21.5) |
| Other[c] | 57 | (8.5) | 3 | (1.0) | 90 | (8.0) | 5 | (0.9) | 75 | (7.6) | 9 | (1.8) |
| Total number of subjects | 667 | (100.0) | 315 | (100.0) | 1119 | (100.0) | 580 | (100.0) | 989 | (100.0) | 497 | (100.0) |

[a]Those who had never smoked as much as one cigarette per day for as long as a year or smoked cigars or a pipe regularly for as long as a year, and who had smoked less in total, than 500 cigarettes, 100 cigars or 20 oz of pipe tobacco. [b]Ex-smokers are those who had stopped smoking at the onset of their illness (lung cancers and hospital controls) or on the date of their interview (population controls). [c]Current pipe or cigar smokers who did not smoke cigarettes and occasional smokers, i.e. those who were not lifelong non-smokers but had never smoked as much as one cigarette per day or cigars/pipe for as long as a year.

**Table 4** Residence in 30-year period ending 5 years before interview

| | Lung cancer | | Hospital controls | | Population controls | |
|---|---|---|---|---|---|---|
| | Male | Female | Male | Female | Male | Female |
| *Years resident in Devon and Cornwall* | | | | | | |
| Mean | 28.73 | 28.97 | 28.79 | 28.78 | 28.76 | 28.72 |
| Standard error | 0.10 | 0.14 | 0.08 | 0.11 | 0.08 | 0.12 |
| Percentage of subjects who lived in Devon or Cornwall for full 30-year period | 76 | 81 | 76 | 78 | 76 | 76 |
| *Number of addresses* | | | | | | |
| Mean | 3.19 | 2.98 | 3.05 | 3.08 | 3.25 | 3.16 |
| Standard error | 0.08 | 0.10 | 0.06 | 0.08 | 0.07 | 0.09 |

one in the living area and one in a bedroom: for the study subjects' current address, this was the subject's own bedroom, while for past addresses it was a bedroom that was currently in use. For subjects who had lived in their current home for more than 5 years, radon detectors were provided by research assistants, who visited the home to check that the detectors had been correctly placed and subsequently retrieved the detectors. Current residents of previous homes of study subjects in Devon and Cornwall were contacted by the National Radiological Protection Board (NRPB) by post and invited to participate in the study. Radon detectors were sent by post to those who agreed, with detailed instructions on installation. Non-responders were sent a second and, if necessary, a third letter. The postal approach was successful for approximately 50% of the current residents of previous homes. When it was not successful, personal visits were made by research assistants.

The small passive radon detectors were manufactured by the NRPB. The production and processing methods conformed to the criteria of a formal validation scheme (Cliff et al. 1991), the accuracy of the measurements was tested every 6 months, and stringent quality assurance procedures were applied (Hardcastle et al. 1996). Each detector consists of a small chamber containing a sensitive plastic material. Radon diffuses into the chamber and decays through its chain of decay products. Some of the alpha particles emitted damage the sensitive plastic element, and this damage is revealed later by etching the plastic in a solution of sodium hydroxide. The etched tracks are counted with an automatic image analyser, and their number is proportional to the exposure of the detector to radon.

The detectors remained in place for 6 months before return to the NRPB for analysis. Precautions were taken to prevent the detectors recording appreciable exposure to radon in the period before and after monitoring in the target address. Before despatch from the NRPB, the detectors were stored in nitrogen, which provides a low-radon environment. Detectors were transported between the NRPB and Devon and Cornwall by post. A typical transit time would be 3 days, and a large proportion of this time would be spent essentially in outdoor air. Outdoor radon levels in the UK are low (Wrixon et al. 1988, Appendix J), and the small percentage of time spent indoors, in places such as sorting offices, is unlikely to have made a material contribution to the overall radon exposure recorded by the detector.

The research assistants were instructed to store detectors for a maximum of 6 weeks before placement in homes and to keep them in a low-radon environment, such as a well-ventilated upstairs room or a vehicle. A control detector was supplied with each batch of detectors, and it remained in the local storage place for the total period during which the detectors in that batch were out in the field. The results from these control detectors provided reassurance that

© Cancer Research Campaign 1998 

**Table 5** Outcome of radon measurement programme in residential addresses occupied by study subjects for at least 1 year

| | Lung cancer | | | | Controls | | | |
|---|---|---|---|---|---|---|---|---|
| | Residential addresses | | Average years of residence per subject | | Residential addresses | | Average years of residence per subject | |
| | No. | (%) | No. | (%) | No. | (%) | No. | (%) |
| *Considering 30-year period ending 5 years before interview* | | | | | | | | |
| Measurement obtained | 2204 | (71.9) | 25.19 | (84.0) | 7244 | (72.7) | 25.52 | (85.1) |
| Concentration assumed low[a] | 32 | (1.0) | 0.12 | (0.4) | 57 | (0.6) | 0.07 | (0.2) |
| Demolished | 147 | (4.8) | 0.97 | (3.2) | 416 | (4.2) | 0.76 | (2.5) |
| Permission withheld | 273 | (8.9) | 2.19 | (7.3) | 864 | (8.7) | 2.23 | (7.4) |
| Not located | 32 | (1.0) | 0.12 | (0.4) | 61 | (0.6) | 0.08 | (0.3) |
| Converted into workplace | 0 | (0.0) | 0.00 | (0.0) | 3 | (0.0) | 0.01 | (0.0) |
| Mobile in Devon/Cornwall[b] | 40 | (1.3) | 0.18 | (0.6) | 112 | (1.1) | 0.10 | (0.3) |
| Other UK | 238 | (7.8) | 0.90 | (3.0) | 882 | (8.9) | 0.91 | (3.0) |
| Non-UK | 90 | (2.9) | 0.29 | (1.0) | 312 | (3.1) | 0.30 | (1.0) |
| At sea | 9 | (0.3) | 0.03 | (0.1) | 11 | (0.1) | 0.01 | (0.0) |
| Total | 3065 | (100.0) | 30.00 | (100.0) | 9962 | (100.0) | 30.00 | (100.0) |
| *Considering 10-year period ending 5 years before interview* | | | | | | | | |
| Measurement obtained | 1249 | (90.0) | 9.23 | (92.3) | 4054 | (90.1) | 9.26 | (92.6) |
| Concentration assumed low[a] | 5 | (0.4) | 0.01 | (0.1) | 10 | (0.2) | 0.02 | (0.2) |
| Demolished | 11 | (0.8) | 0.06 | (0.6) | 34 | (0.8) | 0.06 | (0.6) |
| Permission withheld | 98 | (7.1) | 0.60 | (6.0) | 301 | (6.7) | 0.58 | (5.8) |
| Not located | 4 | (0.3) | 0.01 | (0.1) | 6 | (0.1) | 0.01 | (0.1) |
| Converted into workplace | 0 | (0.0) | 0.00 | (0.0) | 3 | (0.1) | 0.01 | (0.1) |
| Mobile in Devon/Cornwall[b] | 6 | (0.4) | 0.03 | (0.3) | 27 | (0.6) | 0.03 | (0.3) |
| Other UK | 10 | (0.7) | 0.03 | (0.3) | 47 | (1.0) | 0.04 | (0.4) |
| Non-UK | 3 | (0.2) | 0.02 | (0.2) | 15 | (0.3) | 0.01 | (0.1) |
| At sea | 1 | (0.1) | 0.01 | (0.1) | 1 | (0.0) | 0.00 | (0.0) |
| Total | 1387 | (100.0) | 10.00 | (100.0) | 4498 | (100.0) | 10.00 | (100.0) |

[a]Houseboats, caravans, etc. Radon concentration assumed equal to outdoor level of 4 Bq $m^{-3}$. [b]Subject occupied several dwellings for short periods over a total period of more than a year. Each such period is counted as one 'address' in the table.

**Table 6** Distribution of seasonally adjusted radon measurements in dwellings occupied by study subjects during the 30-year period ending 5 years before interview

| Radon gas concentration (Bq $m^{-3}$) | Lung cancer Number of addresses (%) | Controls Number of addresses (%) |
|---|---|---|
| <25 | 1004 (45.6) | 3238 (44.7) |
| 25–49 | 563 (25.5) | 1896 (26.2) |
| 50–99 | 349 (15.8) | 1221 (16.9) |
| 100–199 | 183 (8.3) | 581 (8.0) |
| 200–399 | 67 (3.0) | 204 (2.8) |
| 400–799 | 27 (1.2) | 74 (1.0) |
| 800+ | 11 (0.5) | 30 (0.4) |
| Total number of dwellings measured | 2204 (100.0) | 7244 (100.0) |
| Arithmetic mean[a] | 58 | 56 |
| Quartiles[a] | (15, 28, 57) | (15, 28, 58) |
| Maximum[a] | 1876 | 3549 |

[a]Bq $m^{-3}$.

no material radon exposure occurred during transit to and from the NRPB or in local storage.

The 4167 study subjects had a total of 13 027 relevant residential addresses during the 30-year period of interest (Table 5), and measurements were obtained in 9448 (72.5%) of these addresses. An additional 89 were houseboats, caravans, etc., where the radon concentration would be low and was assumed to be equal to the outdoor level for the UK, estimated by Wrixon et al (1988, Appendix J) to be 4 Bq $m^{-3}$. A further 20 addresses corresponded to periods at sea, where the radon concentration is very low (UNSCEAR, 1982) and was assumed to be zero. When attention was restricted to the 10-year period ending 5 years before interview,

 © Cancer Research Campaign 1998

**Table 7** Distribution of time-weighted average residential radon concentrations experienced by study subjects during the 30-year period ending 5 years before interview, based on measured values and estimates for which no measurement could be obtained. The method of estimation used was that for analyses based on observed values (see section on *Information on residential radon concentrations*).

| Radon gas concentration (Bq m$^{-3}$) | Number of subjects with lung cancer (%) | Number of controls (%) |
|---|---|---|
| <25 | 341 (34.7) | 1084 (34.0) |
| 25–49 | 325 (33.1) | 1111 (34.9) |
| 50–99 | 187 (19.0) | 584 (18.3) |
| 100–199 | 88 (9.0) | 298 (9.4) |
| 200–399 | 32 (3.3) | 85 (2.7) |
| 400–799 | 6 (0.6) | 19 (0.6) |
| 800+ | 3 (0.3) | 4 (0.1) |
| Total number of subjects | 982 (100.0) | 3185 (100.0) |
| Arithmetic mean[a] | 58 | 55 |
| Quartiles[a] | (21, 33, 61) | (21, 33, 61) |
| Maximum[a] | 1700 | 1266 |

[a]Bq m$^{-3}$.

there was a total of 5885 addresses, and measurements were obtained for 5303 (90.1%). The proportions of addresses at which radon measurements were obtained were very similar for subjects with lung cancer and for controls (see Table 5). For 2121 subjects (51%), measurements were obtained for all the relevant addresses.

In order to take account of the possibility that radon remedial measures had been taken at some addresses, current residents of study subjects' past addresses were asked whether any such measures had been taken, and the NRPB's database of approximately 100 000 radon measurements in Devon and Cornwall (NRPB, 1996) was searched for evidence of previous measurements at both current and past addresses of study subjects. For nine addresses, a previous measurement was found that was above the 200 Bq m$^{-3}$ action level (NRPB, 1990) and that was more than twice the more recent measurement. For these nine, it was assumed that remedial measures had been implemented 3 months after the earlier measurement and that the earlier value applied up until then.

For each address at which the radon had been measured, the average annual radon concentration was estimated assuming that 45% of indoor time was spent in the living area and 55% in the bedroom (Wrixon et al, 1988, Appendix M), and using the seasonal correction factors derived by Pinel et al (1995). Each subject's time-weighted average indoor radon concentration was calculated during the 30-year period of interest, when the weights were equal to the number of years spent at each address. The weighted average was based on measured values, when these were available, and on estimated values for addresses when no measurement could be made.

For addresses at which no measurement could be made, estimates of the average annual radon concentration were obtained by different methods for addresses in Devon and Cornwall and for addresses elsewhere. Different estimates were also used according to whether analyses were based on observed radon concentrations ignoring the uncertainties in their assessment, or whether these uncertainties were taken into account.

For analyses based on observed radon values, i.e. ignoring uncertainties in the assessment of radon concentrations, estimation for addresses in Devon or Cornwall was carried out as follows. First, the approximately 100 000 radon measurements in Devon and Cornwall in the NRPB database were examined, and the geometric mean radon concentration for addresses in each 5-km grid square was obtained. The areas of Devon and Cornwall corresponding to the grid squares were then classified into six geographical groups according to whether the mean was <20, 20–32, 33–54, 55–89, 90–147 or ≥ 148 Bq m$^{-3}$ ([i.e. <3.0, 3.0–3.4, 3.5–3.9, 4.0–4.4, 4.5–4.9 or ≥ 5.0 $\log_e$ (Bq m$^{-3}$)], resulting in geographical groups in which radon concentrations were likely to be similar. All the addresses relating to study subjects for whom no measurement could be made were then classified into the same six geographical groups according to the grid square in which they were situated. Measurements obtained specifically for the study tended to be lower than those in the NRPB database. In consequence, the missing values in each group were estimated by considering only measurements made specifically for the study. Estimates were based on measurements for control subjects only, as recommended by Weinberg et al (1996), and because the control subjects were a close approximation to the population from which the study subjects were drawn. The estimates were calculated as the arithmetic mean in each geographical group (Weinberg et al, 1996). This method of estimation was chosen after evaluating the performance of several different methods in predicting the measurements that had been made for the study. Use of a larger number of geographical groups or adjustment for housing characteristics by fitting regression models did not improve prediction performance appreciably.

For study subjects who occupied several addresses in Devon or Cornwall for short periods covering a total period of more than a year, the radon concentration for this period was estimated by the arithmetic mean of all measurements made throughout Devon and Cornwall specifically for control subjects.

Values for UK addresses not in Devon or Cornwall were estimated from the NRPB national database, which includes over 150 000 measurements in areas other than Devon and Cornwall. When the full postcode of the address was available, these were based on the arithmetic mean of the nearest 20 results, and for other addresses a county or other appropriate mean was used. For addresses not in the UK, the world average of 40 Bq m$^{-3}$ was used (UNSCEAR, 1993).

© Cancer Research Campaign 1998 

The methods used for estimating missing radon concentrations in the analyses that took uncertainties into account are described in the Appendix.

### Information on other factors

During the interview, subjects were questioned about smoking habits, occupational history, carotene consumption[1], exposure to radiotherapy and county of birth. Women who were married or widowed were also asked about their husband's current or last occupation. Smoking habits were classified according to consumption at the onset of the illness that brought the subject to hospital (lung cancers and hospital controls) or current consumption (population controls). For current cigarette smokers who also smoked a pipe, tobacco consumption was converted to cigarette equivalents by assuming that 1 oz of pipe tobacco per week was equivalent with regard to the risk of lung cancer to two cigarettes per day (Doll and Peto, 1976) and was added to their cigarette consumption. Few current cigarette smokers also smoked cigars or cigarillos, and no allowance was made for these. Each job held by a study subject for more than a year was classified according to whether or not it was likely to incur a specific risk of lung cancer. Those considered to incur a possible risk were: work underground in a tin or other mine in Devon and Cornwall (Hodgson and Jones, 1990), and jobs with asbestos exposure, including dockyard work (Harries, 1968; Acheson and Gardner, 1979). Subjects were classified into three social class groupings: I & II, III non-manual or manual, and IV & V (Office of Population Censuses and Surveys, 1980) based on their current or last job or, for married women, their husband's current or last job. A list of all foods consumed in the UK that are appreciable sources of carotene was compiled by a nutritionist, and, during the interview, subjects were questioned as to the frequency with which each was consumed. For each subject an estimate of daily carotene consumption was calculated using standard portion sizes obtained from survey and other data (Ministry of Agriculture, Fisheries and Food, 1993) and tables of the composition of foods, in which carotene was expressed in the form of beta-carotene equivalents (Holland et al, 1991). Subjects were then divided into quartiles according to their estimated carotene consumption. For patients who reported that they had received radiotherapy, their statements were assessed by a radiotherapist to determine whether or not they were likely to have caused a dose to the lung of more than 1 gray. For all of these factors, the findings were in the direction expected, and the detailed results will be reported elsewhere.

### Statistical methods

Initial analyses were based on observed radon concentrations, and they ignored uncertainties in the assessment of radon exposure. In these analyses, associations between lung cancer risk and observed time-weighted radon concentrations were studied using the Stata statistical package (StataCorp, 1997). Relative risks are maximum-likelihood values based on unconditional logistic regression with adjustment for age (5-year intervals), sex, smoking status (seven categories, as in Table 3), county of residence and social class (three categories). Estimates of excess relative risk (ERR) per 100 Bq m$^{-3}$ are based on linear logistic regressions and use estimated radon exposure in individual subjects considered as a continuous variable. Inclusion of additional terms in the regression models, such as interactions between the terms listed above, or additional factors, such as place of current residence (individual county district, or urban/rural status of county district), carotene consumption (four categories), work in a job incurring a potential lung cancer risk, exposure to radiotherapy or birth in Devon or Cornwall, altered the estimate of ERR at 100 Bq m$^{-3}$ by 6% at most. Analyses based on linear, as opposed to linear logistic, models of radon risk or conditional on the adjustment variables also gave similar answers. Significance levels are based on the likelihood ratio test, and confidence intervals are based on standard errors. For estimates of relative risks within categories of radon concentration, cutpoints were chosen on the basis of the distribution of time-weighted average radon concentrations for control subjects and without prior knowledge of the relative risks. Heterogeneity tests for subject and tumour characteristics were based on the likelihood ratio. Analyses that took into account uncertainties in the assessment of radon exposure were carried out using the method developed by Reeves et al (1998). Further details are given in the Appendix.

### RESULTS

The arithmetic mean of the seasonally adjusted radon levels in the 9448 addresses at which measurements were obtained was 57 Bq m$^{-3}$. The values were approximately log-normally distributed and the quartiles of the distribution occurred at 15, 28 and 58 Bq m$^{-3}$, while the highest measured concentration was 3549 Bq m$^{-3}$. The distribution of the measurements in subjects with lung cancer and controls was very similar (Table 6).

When the 30-year period of interest was considered for each subject, measurements were available for an average of 25.2 and 25.5 years for subjects with lung cancer and controls, respectively, corresponding to 84.0% and 85.1% of the period of interest (see Table 5). After substitution of estimates for addresses at which no measurement could be obtained, the time-weighted residential radon concentration experienced by subjects with lung cancer during the 30-year period of interest had arithmetic mean 58 Bq m$^{-3}$, while the value for controls was very close at 55 Bq m$^{-3}$ (Table 7). The crude ERR per 100 Bq m$^{-3}$ based on the observed time-weighted radon concentrations for individual subjects was 0.05 (95% CI –0.04, 0.14).

When lung cancer risk was examined in relation to observed time-weighted radon concentration after adjusting for age, sex, smoking status, county of residence and social class, the relative risks in comparison with <25 Bq m$^{-3}$ were 1.06 (95% confidence interval (CI) 0.88, 1.29), 1.13 (95% CI 0.89, 1.44), 0.94 (95% CI 0.68, 1.29), 1.29 (95% CI 0.79, 2.12), and 1.79 (95% CI 0.74, 4.33) for categories 25–49, 50–99, 100–199 and 200–399 and 400+ Bq m$^{-3}$, respectively, and the estimated ERR per 100 Bq m$^{-3}$ based on the observed radon concentrations for individual subjects was 0.08 (95% CI –0.03, 0.20) (Table 8 and Figure 1, top panel). In all subsequent analyses, references to estimated ERRs are to risks estimated as above (i.e. time-weighted and adjusted for these five characteristics) unless otherwise stated.

There is known to be uncertainty in the measurement of residential radon concentrations, as illustrated by the fact that, when the radon concentration in a house is measured on two separate occasions, the values obtained on the two occasions differ (Lomas and Green, 1994), with the ratios having approximately a log-normal distribution

[1]At the time the study was started it was widely believed that carotene was likely to be the agent primarily responsible for the prophylactic value of green and yellow vegetables. This no longer seems likely to be true. Our findings, however, show that it did serve as a good index of whatever benefits the consumption of green and yellow vegetables might produce (to be published).

 © Cancer Research Campaign 1998

**Table 8** Relative risk of lung cancer by various measures of residential radon concentration during the 30-year period ending 5 years before interview. All subjects are included in the analysis (982 cases and 3185 controls)

| | Observed radon concentration (Bq m$^{-3}$) | | | | | | | | | | | | Excess relative risk[e] per 100 Bq m$^{-3}$ | |
|---|---|---|---|---|---|---|---|---|---|---|---|---|---|---|
| **Measure of residential radon concentration** | <25[a] | | 25–49 | | 50–99 | | 100–199 | | 200–399 | | 400+ | | Based on observed values (CI) | Adjusted for uncertainties (CI) |
| | Cases/ Controls[b] | RR[c] | Cases/ Controls | RR (CI)[d] | Cases/ Controls | RR (CI) | Cases/ Controls | RR (CI) | Cases/ Controls | RR (CI) | Cases/ Controls | RR (CI) | | |
| Time-weighted average[f] | 341/ 1084 | 1.00 | 325/ 1111 | 1.06 (0.88,1.29) | 187/ 584 | 1.13 (0.89,1.44) | 88/ 298 | 0.94 (0.68,1.29) | 32/ 85 | 1.29 (0.79,2.12) | 9/ 23 | 1.79 (0.74,4.33) | 0.08 (–0.03,0.20) | 0.12 (–0.05,0.33) |
| Time-weighted average with additional period weighting[g] | 365/ 1103 | 1.00 | 295/ 1083 | 0.94 (0.78,1.14) | 190/ 594 | 1.06 (0.84,1.34) | 88/ 293 | 0.93 (0.68,1.28) | 34/ 87 | 1.24 (0.77,2.01) | 10/ 25 | 1.64 (0.70,3.84) | 0.07 (–0.03,0.19) | 0.11 (–0.06,0.31) |
| Mean time-weighted average radon concentrations | | | | | | | | | | | | | | |
| Based on observed values | 17 | | 35 | | 70 | | 135 | | 259 | | 662 | | | |
| Adjusted for uncertainties | 21 | | 37 | | 67 | | 121 | | 202 | | 371 | | | |

[a]Baseline category. [b]Numbers of cases and controls. [c]Relative risk adjusted for age, sex, smoking status, county of residence and social class. [d]95% Confidence interval. [e]The increase in relative risk per 100 Bq m$^{-3}$ increase in radon concentration. [f]Radon concentration for each address weighted according to the length of time that the subject lived there. [g]Periods 5–14, 15–24 and 25–34 years before interview weighted in proportions 1.0:0.75:0.50.

**Figure 1** Relative risk of lung cancer according to residential radon concentration adjusted for age, sex, smoking status, county of residence and social class. In the top panel, relative risks and 95% confidence intervals are shown by mean time-weighted average concentration during the 30-year period ending 5 years before interview for individuals with observed values in categories <25, 25–49, 50–99, 100–199, 200–399 and 400+ Bq m$^{-3}$, and the fitted regression line is based on observed values for individual subjects. In the bottom panel, the mean values and fitted regression line have been adjusted for uncertainties in the assessment of radon concentration. In the top panel the fitted regression line corresponds to an ERR per 100 Bq m$^{-3}$ of 0.08, while for the bottom panel the value is 0.12

(Reeves et al, 1998). As a consequence of this uncertainty, observed radon concentrations at the upper end of the distribution will tend to be considerably higher than their true values, while observed radon concentrations at the lower end of the distribution will tend to be slightly lower than their true values, and ERRs based on observed radon concentrations will underestimate any risk (Cox et al, 1998). When the methods described in the Appendix were used to adjust for uncertainties in the assessment of radon exposure, the mean values of the true time-weighted average radon concentration for individuals whose observed values lay in the categories 200–399 and 400+ Bq m$^{-3}$ were estimated to be 202 and 371 Bq m$^{-3}$, respectively, considerably lower than their observed values of 259 and 662 Bq m$^{-3}$ (see Table 8), and the estimated ERR per 100 Bq m$^{-3}$ after adjusting for uncertainty was 0.12 (95% CI –0.05, 0.33) (see Figure 1). This estimate is larger, and also has a wider confidence interval, than the estimate based on observed radon concentrations, i.e. without allowing for uncertainty.

Studies of patterns of lung cancer in underground miners exposed to high levels of radon have suggested that exposure during the previous 5–15 years carries a greater risk per unit exposure than that received in the more distant past (see, for example, Tomasek et al, 1994). When the present analysis was repeated considering radon concentrations during the 30-year period ending 5 years before the interview, but weighting the exposure received during periods 5–14, 15–24 and 25–34 years previously in proportions 1.0:0.75:0.50, as suggested by recent analyses of data from the studies of miners (Lubin et al, 1997), the ERR based on the observed radon concentrations was 0.07 (95% CI –0.03, 0.19), while the corresponding estimate after adjusting for uncertainties in the assessment of radon exposure was 0.11 (95% CI –0.06, 0.31; see Table 8). Both these estimates are very similar to the values obtained when all time periods were weighted equally.

When the analysis was limited to the 2121 subjects (49% of those with lung cancer and 51% of the controls) for whom measurements were available for all 30 years, the ERR per 100 Bq m$^{-3}$ based on observed radon concentrations was 0.14 (95% CI 0.01, 0.29), somewhat greater than the value obtained when all subjects were included in the analysis, although the difference between the two groups was

**Table 9** Relative risk of lung cancer by various measures of residential radon concentration during the 30-year period ending 5 years before interview. Only subjects with radon measurements available for all 30 years are included in the analysis (484 cases and 1637 controls)

| Measure of residential radon concentration | Observed radon concentration (Bq m$^{-3}$) <25[a] Cases/ Controls[b] | <25[a] RR[c] | 25–49 Cases/ Controls | 25–49 RR (CI)[d] | 50–99 Cases/ Controls | 50–99 RR (CI) | 100–199 Cases/ Controls | 100–199 RR (CI) | 200–399 Cases/ Controls | 200–399 RR (CI) | 400+ Cases/ Controls | 400+ RR (CI) | Excess relative risk[e] per 100 Bq m$^{-3}$ Based on observed values (CI) | Excess relative risk[e] per 100 Bq m$^{-3}$ Adjusted for uncertainties (CI) |
|---|---|---|---|---|---|---|---|---|---|---|---|---|---|---|
| Time-weighted average[e] | 194/ 660 | 1.00 | 136/ 496 | 1.11 (0.84.1.46) | 92/ 276 | 1.45 (1.03.2.02) | 41/ 145 | 0.98 (0.63.1.54) | 15/ 45 | 1.15 (0.56.2.37) | 6/ 15 | 3.12 (1.07.9.04) | 0.14 (0.01.0.29) | 0.24 (–0.01.0.56) |
| Time-weighted average with additional period weighting[f] | 200/ 664 | 1.00 | 128/ 484 | 1.05 (0.79.1.40) | 93 292 | 1.31 (0.94.1.82) | 40/ 137 | 1.07 (0.68.1.68) | 17/ 44 | 1.35 (0.68.2.70) | 6/ 16 | 2.48 (0.85.7.24) | 0.14 (0.01.0.28) | 0.24 (–0.01.0.55) |
| Mean time-weighted average radon concentrations | | | | | | | | | | | | | | |
| Based on observed values | 16 | | 35 | | 69 | | 133 | | 266 | | 703 | | | |
| Adjusted for uncertainties | 20 | | 36 | | 62 | | 106 | | 184 | | 384 | | | |

[a]Baseline category. [b]Numbers of cases and controls. [c]Relative risk adjusted for age, sex, smoking status, county of residence and social class. [d]95% Confidence interval. [e]Radon concentration for each address weighted according to the length of time that the subject lived there. [f]Periods 5–14, 15–24 and 25–34 years before interview weighted in proportions 1.0:0.75:0.50.

**Table 10** Excess relative risks (ERR) and standard errors (s.e.) of lung cancer per 100 Bq m$^{-3}$ based on time-weighted average observed residential radon concentrations during the 30-year period ending 5 years before interview for various tumour characteristics

| Tumour characteristic | Cases/Controls | Excess relative risk per 100 Bq m$^{-3}$ based on observed radon concentration: ERR (s.e.) | ERR (with CI) |
|---|---|---|---|
| Histological type | | | |
| Squamous cell | 332/3185 | –0.05 (0.09) | |
| Small cell | 192/3185 | 0.20 (0.09) | |
| Adenocarcinoma | 77/3185 | 0.18 (0.14) | |
| Other | 235/3185 | 0.03 (0.11) | |
| No microscopic evidence | 146/3185 | –0.02 (0.14) | |
| Test for heterogeneity $\chi^2_4$ = 4.9. $P$ = 0.29 | | | |
| Site of tumour | | | |
| Main bronchus | 231/3185 | –0.07 (0.12) | |
| Other | 751/3185 | 0.10 (0.06) | |
| Test for heterogeneity $\chi^2_1$ = 2.2. $P$ = 0.13 | | | |
| All subjects | 982/3185 | 0.08 (0.06) | |

Each analysis is adjusted for age, sex, smoking status, county of residence and social class. For subgroups, black squares indicate ERRs and have area inversely proportional to the variance of the ERR, i.e. proportional to the amount of information contributed, while horizontal lines indicate 99% confidence intervals (CI). For all subjects, the diamond has height proportional to the square root of the amount of information contributed and width equal to the 95% CI. The solid vertical line represents an ERR of 0.0 and the broken vertical line indicates the ERR for all subjects.

not significant statistically (heterogeneity test $\chi^2_1$ = 1.3, $P$ = 0.26). After adjusting for uncertainties, the estimated ERR per 100 Bq m$^{-3}$ increased further to 0.24 (95% CI –0.01, 0.56). As for the analysis when all subjects were included, additional period-weighting to give greater weight to exposure in the more recent past changed the estimate of risk by very little (Table 9).

To see whether there was any evidence that the effect of radon differed according to the characteristics of the tumour, the ERRs per 100 Bq m$^{-3}$ based on observed radon concentrations were estimated separately by histological type and by site of tumour (Table 10). Although there was some variation in the ERRs, with higher values occurring for small-cell tumours than for other histological types, and for tumours beyond the main bronchi, including the periphery of the lung, rather than in the main bronchi, the observed variations were not greater than would be expected by chance (histological type $\chi^2_4$ = 4.9, $P$ = 0.29; site of tumour $\chi^2_1$ = 2.2, $P$ = 0.13). When tumours for which no microscopic evidence was available were excluded, the ERR per 100 Bq m$^{-3}$ for the remaining tumours was 0.09 (95% CI –0.02, 0.21), very similar to the value of 0.08 (95% CI –0.03, 0.20) obtained when they were included.

 © Cancer Research Campaign 1998

**Table 11** Excess relative risks (ERR) and standard errors (s.e.) of lung cancer per 100 Bq m$^{-3}$ based on time-weighted average observed residential radon concentrations during the 30-year period ending 5 years before interview for various subject characteristics. Analyses are adjusted for age, sex, smoking status, county and social class

| Subject characteristic | Cases/Controls | Excess relative risk per 100 Bq m$^{-3}$ based on observed radon concentration: ERR (s.e.) | ERR (with CI) |
|---|---|---|---|
| Sex | | | |
| Male | 667/2108 | 0.14 (0.07) | |
| Female | 315/1077 | –0.18 (0.11) | |
| Test for heterogeneity $\chi^2_1$ = 3.7, $P$ = 0.05 | | | |
| Age (years) | | | |
| <55 | 99/413 | 0.31 (0.36) | |
| 55–64 | 297/1017 | –0.06 (0.12) | |
| 65–76 | 586/1755 | 0.10 (0.07) | |
| Test for heterogeneity $\chi^2_2$ = 2.1, $P$ = 0.36 | | | |
| Smoking status | | | |
| Lifelong non-smoker | 26/913 | 0.04 (0.27) | |
| Current cigarette smoker | 517/670 | –0.04 (0.09) | |
| Ex-smoker | 379/1423 | 0.19 (0.08) | |
| Other | 60/179 | –0.23 (0.24) | |
| Test for heterogeneity $\chi^2_3$ = 2.8, $P$ = 0.42 | | | |
| Years working outdoors | | | |
| 0 | 543/1795 | –0.03 (0.08) | |
| 1–20 | 200/634 | 0.12 (0.16) | |
| 21+ | 239/756 | 0.22 (0.11) | |
| Test for heterogeneity $\chi^2_2$ = 2.0, $P$ = 0.36 | | | |
| All subjects | 982/3185 | 0.08 (0.06) | |

The youngest subject with lung cancer was aged 30 years while 25 subjects (two with lung cancer and 23 controls) were aged 74 years when selected but were aged 75 years (24 subjects) or 76 years (one control) at interview. The ERR for current cigarette smokers is adjusted for amount smoked in categories <15, 15–24 and 25+ cigarettes per day. The ERR for ex-smokers is adjusted for time since quitting in categories <10 years and 10+ years. Other smokers are current pipe or cigar smokers who do not smoke cigarettes, and occasional smokers. Years working outdoors are full-time equivalent years in the 30-year period ending 5 years before interview. Years of part-time work are counted pro-rata. Symbols and other details are as in Table 10.

A similar analysis was carried out to see whether there was any evidence that the effect of radon differed according to any known characteristics of the subject (Table 11). Out of the four characteristics considered (sex, age, smoking status and years spent working outdoors), there was evidence of heterogeneity only for sex ($\chi^2_1$ = 3.7, $P$ = 0.05), with women having a lower ERR per 100 Bq m$^{-3}$ than men. Among the remaining categories, ERRs were highest for subjects aged less than 55 years, for ex-smokers and for those who had worked out of doors for more than 20 years, but there was no evidence of heterogeneity for any of these characteristics (see Table 11).

### DISCUSSION

This report presents the results of a large, population-based study specifically designed to examine the relationship between residential radon concentration and lung cancer risk. The study was carried out in the part of the UK where the highest concentrations of residential radon occur and, in order to identify a group of people likely to have been exposed to high average concentrations during the previous 35 years, was restricted to long-term residents of the area. To ensure that the subjects with lung cancer included in the study represented as closely as possible those occurring in the study population, only incident cases were included and, to minimize any biases in the information on smoking and factors other than residential radon that determine lung cancer risk, all study subjects were personally interviewed by trained research assistants using standard questionnaires.

The exposure of interest is the residential radon concentration experienced by the study subjects in the past. This cannot be assessed directly, as it is possible only to measure current concentrations in both current and previous residences. There is, however, evidence from a study of temporal variations in residential radon concentrations that, in the high radon areas of the UK, levels have not increased appreciably in general, at least during the decade before this study (Lomas and Green, 1994). In addition, efforts were made in the present study to identify any dwellings occupied by study subjects where radon remedial measures were likely to have been taken and to estimate the radon concentrations appropriately.

Radon concentrations found in the present study were lower than those found in the NRPB's large database of approximately 100 000 measurements within Devon and Cornwall. This is chiefly accounted for by a tendency for the dwellings included in the NRPB database to be preferentially located in the highest radon areas within Devon and Cornwall. An earlier survey by the NRPB of radon concentrations in UK residential addresses selected to be representative of the whole country from files maintained by the Post Office included 37 measurements for Devon and 16 for Cornwall and gave arithmetic means of 72 (95% CI 19, 125)

and 114 (95% CI 67, 162) for the two counties respectively. The corresponding values in the present study for addresses occupied by control subjects were 42 (95% CI 40, 44) for Devon and 108 (95% CI 100, 116) for Cornwall, based on 5706 and 1538 measurements respectively. The values observed in the present study are therefore consistent with those observed in the NRPB representative survey.

Although strenuous attempts were made to measure the radon concentrations at the addresses of all study subjects during the 30-year period of interest, there were inevitably gaps in the measurement histories, corresponding to 15% of the period of interest, and estimates for these addresses were therefore constructed using a validated methodology (Weinberg et al, 1996) which took into account the location of the address. When the analysis was limited to individuals for whom it was possible to obtain radon measurements for the entire 30-year period of interest, the estimated excess relative risks were larger than for the entire group. This may be a chance finding or it may be a reflection of the fact that more information is available regarding the exposure histories in this subgroup.

Radon concentrations that have been estimated rather than measured are inevitably subject to uncertainty. Measured radon concentrations are, however, also subject to uncertainty in the sense that, when a dwelling is measured twice, values that differ appreciably will usually arise, even when high-quality long-term measurements are carried out and appropriate seasonal corrections applied. A study carried out by the same laboratory as that in the present study, and using similar techniques, indicated a coefficient of variation for repeated measurements in the same house of around 50% (Lomas and Green, 1994). Unless taken into account, this measurement variability will distort the results, in that the highest observed radon concentrations will tend to be overestimates of their true values, and the lowest will tend to be underestimates, so that regression coefficients based on the observed radon concentrations will tend to underestimate the strength of any relationship between true radon concentration and risk of lung cancer, with the extent of the attenuation depending on the size of the measurement variability. Special methodology has therefore been developed that takes appropriate account of the uncertainties due to both measurement and estimation variability in the assessment of time-weighted average radon concentrations (Reeves et al, 1998). In the present study, the relationship between radon concentration and risk of lung cancer has been estimated twice, first in the standard way based on the observed radon concentrations and then after taking the uncertainties into account. The effect of taking account of the uncertainties was to increase both the magnitude of the estimated radon-related risk and the size of the associated confidence interval. Estimates based on observed radon concentrations are appropriate for comparison with the results of other studies of residential radon in which similar uncertainties are likely to be present but have not been taken into account; while estimates in which the uncertainties have been taken into account are more appropriate for comparison with risk estimates derived in different ways and when considering the amount of lung cancer likely to be caused by residential radon.

The risk of lung cancer is determined by other factors as well as residential radon concentration. In the present analysis, logistic regression has been used to adjust for the effects of these factors, the most important of which is smoking status. In order to be sure that no appreciable residual confounding with smoking status remains, seven categories of smoking status have been used in the adjustment, with life-long non-smokers and ex-smokers of durations <10 and 10+ years in separate categories, and three separate categories for current smokers of cigarettes (see Table 3). Previous studies have demonstrated that very little residual confounding remains after this degree of stratification for cigarette consumption (Breslow and Day, 1980). Errors in the assessment of smoking status are also likely to be present. It would be possible from the theoretical point of view to take them into account, but there are few data available with which to quantify such errors. In any case, as there is little confounding between radon and smoking status in the present study, adjustment for errors in the assessment of smoking status would have little effect on the estimated risk from radon.

At the present time, nine case–control studies of indoor radon and lung cancer have been carried out that have each included at least 200 subjects with lung cancer and measured at least one residence for most subjects. These studies have been carried out in Canada, China, Finland, Sweden, the USA and western Germany (Blot et al, 1990; Schoenberg et al, 1990; Pershagen et al, 1992; Alavanja et al, 1994; Letourneau et al, 1994; Pershagen et al, 1994; Auvinen et al, 1996; Ruosteenoja et al, 1996; Wichmann et al, 1997). For eight of these studies, the published relative risks after adjusting for confounding variables have been combined using weighted linear regression to give an estimated excess relative risk of 0.09 (95% CI 0.0, 0.2) per 100 Bq $m^{-3}$ based on observed radon concentrations (Lubin and Boice, 1997), while for the study in western Germany (Wichmann et al, 1997) the estimated excess relative risk per 100 Bq $m^{-3}$ in radon-prone areas based on observed radon concentrations is 0.13 (95% CI –0.12, 0.46). The totality of the evidence from other studies of residential radon and lung cancer therefore suggests an excess relative risk of around 0.1 per 100 Bq $m^{-3}$, based on observed radon concentrations. Thus, the estimated excess relative risk based on observed radon concentrations in the present study of 0.08 per 100 Bq $m^{-3}$ (95% CI –0.03, 0.20) is in close accordance with the findings from other studies. Although the 95% confidence interval for the excess relative risk in the present study just includes zero, the combined evidence suggests that a zero effect would be an inappropriate interpretation of the study results.

The impact of measurement variability on the excess relative risk has been assessed for only one of the nine previous studies (Lagarde et al, 1997). For that study it was also concluded that a coefficient of variation for repeated measurements in the same house was of the order of 50%, and that the excess relative risk of 0.10 per 100 Bq $m^{-3}$ based on the observed concentrations should be corrected to about 0.15–0.20 per 100 Bq $m^{-3}$ when measurement variability was taken into account. This conclusion is very similar to that of the present study, in which accounting for uncertainties increased the estimated relative risk per 100 Bq $m^{-3}$ from 0.08 to 0.12 (95% CI –0.05, 0.33).

The results of the ten studies of the effects of residential radon that are based on individual data together provide strong empirical evidence that the results of ecological regressions, whereby lung cancer rates in geographical areas are related to area-specific average residential radon level and in which a significant negative relationship between residential radon and lung cancer has often been observed, are highly misleading (see for example Piantadosi et al, 1988; Stidley and Samet, 1993; Cohen, 1995; Lubin, 1998).

The findings from the studies of residential radon that are based on individual data are also consistent with the findings from a pooled analysis of 11 studies of underground miners occupationally exposed to radon (Lubin et al, 1995*a*). For miners exposed to, at most, 50 working-level months, which would result in approximately the same bronchial dose as living in a house with a radon

© Cancer Research Campaign 1998

concentration of around 400 Bq $m^{-3}$ for 30 years, an excess relative risk of 0.09 per 100 Bq $m^{-3}$ has been estimated based on 468 deaths (Lubin and Boice, 1997; Lubin et al, 1997). When miners receiving higher exposures were also included in the analysis, a somewhat lower estimate was observed (Lubin et al, 1995*a*), corresponding to an excess relative risk of around 0.05 per 100 Bq $m^{-3}$. For miners exposed to more than about 50 working-level months, an inverse dose-rate effect has been observed, whereby, for a fixed total exposure, greater risks are associated with exposures occurring at a low exposure rate and spread over a long duration than for exposures occurring at a high exposure rate with short duration (Darby and Doll, 1990; Lubin et al, 1995*b*). The inverse dose-rate is likely to occur for exposure levels at which lung epithelial cells are likely to be traversed by more than one alpha particle. Multiple alpha particle traversals are likely to occur in heavily exposed miners, but are rare within the range of radon concentrations usually experienced residentially (National Research Council, 1998). The risks of residential radon exposure are therefore unlikely to be affected by the inverse dose rate effect.

Analyses of mortality patterns in underground miners receiving substantially higher cumulative exposures than would normally occur residentially have demonstrated a tendency for exposures received in the previous 5–15 years to carry a greater risk than exposures received in the more distant past. Moreover, such analyses have also shown that the relative risk associated with a given level of exposure tends to be higher in younger subjects and among non-smokers compared with smokers (Roscoe et al, 1989; Tomasek et al, 1994; Lubin et al, 1997). In addition, there is considerable evidence that relative risks for small-cell cancers are higher than for other histological types of cancer (National Research Council, 1998). In the present study, there was no evidence to suggest that a higher risk of lung cancer is associated with exposure received in the more recent past (Table 8). However, there is little power of discrimination in a study such as this, in which a large proportion of subjects with the highest observed radon concentrations during the 30-year period of interest had lived at the same address for most of the period. Tests for heterogeneity between tumour and subject characteristics suggested a difference in risk only between men and women (Table 11). This was unexpected, and the result may be due to random variation: the chance of finding one out of six independent heterogeneity tests to be significant at a nominal level of 5% when in fact no heterogeneity is present is approximately one in four. Conversely, some of the variation observed between the other subgroups may represent real differences that are not statistically significant because of the limited power of the study. For example, a higher excess relative risk was seen for small-cell tumours than for other types of lung cancer and a higher relative risk was seen in those aged under 55 years than in older subjects. Both of these results would be predicted from the studies of miners receiving much higher exposures. In addition, the higher risks associated with tumours outside the main bronchus may be a result of radon progeny being more liable to be deposited peripherally than in the main airways; and the tendency for the excess relative risk to increase with increasing number of years spent working outdoors may be a reflection of the fact that the time-weighted average radon concentration has been more accurately estimated for these individuals. Nothing of value can, however, be learnt from the interaction with smoking as the number of lung cancers in life-long non-smokers (26) was very small.

Although this study was large in size, with nearly 1000 cases of lung cancer and over 3000 controls, and was carried out in the area of the UK where the highest residential radon concentrations are found, as well as having a highly effective measurements programme covering on average 85% of the 30-year period of interest, it has only limited power to assess the risk associated with residential radon. Plans are in hand for formal pooled analyses of the data from both European and North American studies of lung cancer and residential radon. When these analyses are complete, a more precise estimate of the lung cancer risk should be available, together with clearer evidence on any variation in risk with subject and tumour characteristics.

### CONCLUSION

In the present study, the estimated excess relative risk associated with a 100 Bq $m^{-3}$ increase in residential radon concentration is 0.08 (95% confidence interval –0.03, 0.20) when uncertainties in the assessment of radon exposure are ignored and is 0.12 (95% confidence interval –0.05, 0.33) when these uncertainties are taken into account. Although the confidence intervals for these estimates just include zero, the estimates are similar in magnitude to those derived from other studies of residential radon in which data have been collected on individual subjects, and also from studies of underground miners occupationally exposed at low concentrations. The combined evidence therefore suggests that a zero effect would not be an appropriate interpretation of the study's results and that there is a risk of lung cancer associated with residential radon exposure of about the size that has been postulated on the basis of studies of miners occupationally exposed to radon.

### ACKNOWLEDGEMENTS

Data collection for this study was carried out by the following research assistants: Yvonne Ballard, Susan Callen, Valerie Chapman, Jacqueline Griffith, Susan Newby, Beverley Pinnock, Sally Reid, Kate Sayers, Jane Smithard, Patricia Stunden, Elizabeth Stanley, Siân Whitehead and Martin Whild. Clerical and secretarial assistance was provided by Lindsay Cutler, Isabel Sutherland and Valerie Weare. We thank the subjects who took part in the study and gratefully acknowledge the assistance given to us by the staff in hospitals and general practices throughout Devon and Cornwall. We should also like to thank the Department of Health Steering Committee (chairman Dr William Maton-Howarth) for their encouragement throughout the study, Dr Alistair Laing for reviewing the subjects' responses regarding exposure to radiotherapy, Sir David Cox and Professor Valerie Beral for helpful discussions, and Mr Paul Appleby, Mr Harz Deo and Dr Gerry Kendall.

This study was funded by the Imperial Cancer Research Fund, the National Radiological Protection Board, the Department of Health, the Department of the Environment, Transport and the Regions, and the European Commission.

### REFERENCES

Acheson ED and Gardner MJ (1979) The ill effects of asbestos on health. In *Asbestos: Final Report of the Advisory Committee* vol. 2. HMSO: London

Alavanja MCR, Brownson RC, Lubin JH, Berger E, Chang J and Boice JD Jr (1994) Residential radon exposure and lung cancer among nonsmoking women. *J Natl Cancer Inst* **86**: 1829–1837

Auvinen A, Mäkeläinen I, Hakama M, Castrén O, Pukkala E, Reisbacka H and Rytömaa T (1996) Indoor radon exposure and risk of lung cancer: a nested case–control study in Finland. *J Natl Cancer Inst* **88**: 966–972 (see also Erratum *J Natl Cancer Inst* **90**: 401–402)

Blot WJ, Xu Z-Y, Boice JD Jr, Zhao D-Z, Stone BJ, Sun J, Jing L-B and Fraumeni JF Jr (1990) Indoor radon and lung cancer in China. *J Natl Cancer Inst* **82**: 1025–1030

Breslow NE and Day NE (1980) *Statistical Methods in Cancer Research Vol. I: The Analysis of Case–control Studies*. International Agency for Research on Cancer, Lyon

Clarke RH and Southwood TR (1989) Risks from ionizing radiation. *Nature* **338**: 197–198

Cliff KD, Miles JC and O'Riordan MC (1991) Validation scheme for laboratories making measurements of radon in dwellings. *National Radiological Protection Board memorandum NRPB-M276*. HMSO: London

Cohen BL (1995) Test of the linear no threshold theory of carcinogenesis for inhaled radon decay products. *Health Phys* **68**: 157–174

Cox DR, Darby SC, Reeves GK and Whitley E (1998) The effects of measurement errors with particular reference to a study of exposure to residential radon. In *National Cancer Institute Monograph on Uncertainties in Radiation Dosimetry and their Impact on Dose–Response Analysis* (in press)

Cross FT (1994) Residential radon risks from the perspective of experimental animal studies. *Am J Epidemiol* **140**: 333–339

Darby SC and Doll R (1990) Radiation and exposure rate. *Nature* **344**: 824

Doll R and Peto R (1976) Mortality in relation to smoking: 20 years' observations on male British doctors. *Br Med J* **2**: 1525–1536

Gunby JA, Darby SC, Miles JC, Green BM and Cox DR (1993) Factors affecting indoor radon concentrations in the United Kingdom. *Health Phys* **64**: 2–12

Hardcastle GD, Howarth CB, Naismith SP, Algar RA and Miles JC (1996) NRPB etched-tracked detectors for area monitoring of radon. In *National Radiological Protection Board report NRPB-R283*. HMSO: London

Harries PG (1968) Asbestos hazards in naval dockyards. *Ann Occup Hyg* **11**: 135–145

Hodgson JT and Jones RD (1990) Mortality of a cohort of tin miners 1941–86. *Br J Ind Med* **47**: 665–676

Holland B, Welch AA, Unwin ID, Buss DH, Paul AA and Southgate DAT (1991) *McCance and Widdowson's The Composition of Foods*. 5th edn. Royal Society of Chemistry: Cambridge

International Agency for Research on Cancer (1988) *IARC Monographs on the Evaluation of Carcinogenic Risks to Humans. Vol. 43. Man-made Mineral Fibres and Radon*. IARC: Lyon

Lagarde F, Pershagen G, Åkerblom G, Axelson O, Bäverstam U, Damber L, Enflo A, Svartengren M and Swedjemark GA (1997) Residential radon and lung cancer in Sweden: risk analysis accounting for random error in the exposure assessment. *Health Phys* **72**: 269–276

Létourneau EG, Krewski D, Choi NW, Goddard MJ, McGregor RG, Zielinski JM and Du J (1994) Case–control study of residential radon and lung cancer in Winnipeg, Manitoba, Canada. *Am J Epidemiol* **140**: 310–322

Lomas PR and Green BMR (1994) Temporal variations of radon levels in dwellings. *Radiat Protect Dosim* **56**: 323–325

Lubin J (1998) On the discrepancy between epidemiologic studies in individuals of lung cancer and residential radon and Cohen's ecologic regression. *Health Phys* (in press)

Lubin JH and Boice JD Jr (1997) Lung cancer risk from residential radon: meta analysis of eight epidemiologic studies. *J Natl Cancer Inst* **89**: 49–57

Lubin JH, Boice JD Jr, Edling C, Hornung RW, Howe GR, Kunz E, Kusiak RA, Morrison HI, Radford EP, Samet JM, Tirmarche M, Woodward A, Yao SX and Pierce DA (1995*a*) Lung cancer in radon-exposed miners and estimation of risk from indoor exposure. *J Natl Cancer Inst* **87**: 817–827

Lubin JH, Boice JD Jr., Edling C, Hornung RW, Howe GR, Kunz E, Kusiak RA, Morrison HI, Radford EP, Samet JM, Tirmarche M, Woodward A and Yao SX (1995*b*) Radon-exposed underground miners and inverse dose-rate (protraction enhancement) effects. *Health Phys* **69**: 494–500

Lubin JH, Tomasek L, Edling C, Hornung RW, Howe G, Kunz E, Kusiak RA, Morrison HI, Radford EP, Samet JM, Tirmarche M, Woodward A and Yao SX (1997) Estimating lung cancer mortality from residential radon using data for low exposures of miners. *Radiat Res* **147**: 126–134

Ministry of Agriculture, Fisheries and Food (1993) *Food Portion Sizes*. 2nd edn. HMSO: London

National Radiological Protection Board (1990) Human exposure to radon in homes. In *Documents of the NRPB*, Vol. I, part 1, pp. 17–32. HMSO: London

National Radiological Protection Board (1996) Radon affected areas: England and Wales. In *Documents of the NRPB*, Vol. 7, part 2, pp. 1–18. HMSO: London

National Research Council, Committee on Biological Effects of Ionising Radiation (1998) *Health Risks of Exposure to Radon (BEIR VI)*. National Academy Press: Washington, DC

Office of Population Censuses and Surveys (1980) *Classification of Occupations*. HMSO: London

O'Riordan MC (1993) Protection against natural radiation: achievements and aspirations. In *Radiation Protection on the Threshold of the 21st Century*, pp. 123–134. OECD: Paris

Pershagen G, Liang Z-H, Hrubec Z, Svensson C and Boice JD Jr (1992) Residential radon exposure and lung cancer in Swedish women. *Health Phys* **63**: 179–186

Pershagen G, Åkerblom G, Axelson O, Clavensjö B, Damber L, Desai G, Enflo A, Lagarde F, Mellander H, Svartengren M and Swedjemark GA (1994) Residential radon exposure and lung cancer in Sweden. *N Engl J Med* **330**: 159–164

Piantadosi S, Byar DP and Green SB (1988) The ecological fallacy. *Am J Epidemiol* **127**: 893–904

Pinel J, Fearn T, Darby SC and Miles JC (1995) Seasonal correction factors for indoor radon measurements in the United Kingdom. *Radiat Protect Dosim* **58**: 127–132

Reeves GK, Cox DR, Darby SC and Whitley E (1998) Some aspects of measurement error in explanatory variables for continuous and binary regression models. *Stat Med* (in press)

Roscoe RJ, Steenland K, Halperin WE, Beaumont JJ and Waxweiler RJ (1989) Lung cancer mortality among nonsmoking uranium miners exposed to radon daughters. *JAMA* **262**: 629–633

Ruosteenoja E, Mäkeläinen I, Rytömaa T, Hakulinen T and Hakama M (1996) Radon and lung cancer in Finland. *Health Phys* **71**: 185–189

Schoenberg JB, Klotz JB, Wilcox HB, Nicholls GP, Gil-del-Real MT, Stemhagen A and Mason TJ (1990) Case–control study of residential radon and lung cancer among New Jersey women. *Cancer Res* **50**: 6520–6524

StataCorp (1997) *Stata Statistical Software: Release 5.0*. Stata Corporation: College Station, TX

Stidley CA and Samet JM (1993) A review of ecological studies of lung cancer and indoor radon. *Health Phys* **65**: 234–251

Tomasek L, Darby SC, Fearn T, Swerdlow AJ, Placek P and Kunz E (1994) Patterns of lung cancer mortality among uranium miners in West Bohemia with varying rates of exposure to radon and its progeny. *Radiat Res* **137**: 251–261

United Nations Scientific Committee on the Effects of Atomic Radiation (1982) *Ionizing Radiation: Sources and Biological Effects*. United Nations: New York

United Nations Scientific Committee on the Effects of Atomic Radiation (1993) *Sources and Effects of Ionizing Radiation*. United Nations: New York

Weinberg CR, Moledor ES, Umbach DM and Sandler DP (1996) Imputation for exposure histories with gaps under an excess relative risk model. *Epidemiology* **7**: 490–497

Wichmann HE, Kreienbrock L, Kreuzer M, Gerken M, Dingerkus G, Wellmann J, Keller G and Kappel RJA (1997) Lungenkrebsrisiko durch Radon in der Bundesrepublik Deutschland (West). *Bericht an das Bundesamt für Strahlenschutz und den Bundesminister für Umwelt, Naturschutz und Reaktorsicherheit im Vorhaben St Sch 1066, 4074, 4074/1*. Wuppertal

World Health Organization (1975) *ICD-9. International Classification of Diseases*. WHO: Geneva

World Health Organization (1976) *ICD-0. International Classification of Diseases for Oncology*. WHO: Geneva

Wrixon AD, Green BM, Lomas PR, Miles JC, Cliff KD, Francis EA, Driscoll CM, James AC and O'Riordan MC (1988) Natural radiation exposure in UK dwellings. In *National Radiological Protection Board report NRPB-R190*. HMSO: London

### APPENDIX

### Method of analysis accounting for uncertainties in the assessment of radon exposure

#### The model

Analyses that took into account uncertainties in the assessment of radon exposure were based on the methodology of Reeves et al (1998) and used the following model:

$$\Pr(Y = 1 | X_{s(1)} \ldots X_{s(J)}) = \Lambda\left(\frac{\alpha_t + \beta_t \sum w_j v^*_j X_{s(j)}^{\gamma_{t,s(j)}} + \sum \eta_k Z_k}{\{1 + k^2 \beta_t^2 \sum w_j^2 (v^*_j)^2 X_{s(j)}^{2\gamma_{t,s(j)}} (e^{\sigma^2_{t,s(j)}} - 1)\}^{1/2}}\right) \quad (1)$$

© Cancer Research Campaign 1998

where $Y$ is the binary response variable. $\Lambda(x) = e^x/(1 + e^x)$ is the logistic function, $\alpha_t$ and $\beta_t$ are the intercept and slope, respectively, of the relationship between the logarithm of disease odds and the true residential radon concentration, the index j runs over the addresses belonging to a particular subject, $w_j$ is the weight given to each address and usually represents the proportion of the 30-year period lived at each address ($\Sigma w_j = 1$), the $z_k$ are dummy variables representing the different levels of the covariates (age, sex, smoking status, etc), $\eta_k$ are their associated regression coefficients, and $k = 0.588$ is a multiplicative constant that arises when approximating the logistic by the probit function. $X_{s1}, \ldots X_{sJ}$, are the surrogate (i.e. observed) values of residential radon for the subject, and these and the remaining quantities in equation (1) differ according to whether or not a measurement is available for a particular address and are explained in the following two sections.

### *Addresses for which a measurement was available*

When the measured radon concentration at the jth address of a subject was available, $X_{sj}$, was set equal to it in equation (1). The remaining quantities in equation (1) involve the measurement error variance and the mean and variance of the distributions from which the log radon measurements, i.e. the $\log_e X_{sj}$, are drawn. It is the relationship between these parameters that determines the extent to which the measurement errors affect the estimated relationship. If the log radon measurements are drawn from a distribution with mean $\mu$ and variance $\sigma_t^2$, and the variance of the logs of repeat measurements at the same address is $\sigma_m^2$, then from Reeves et al (1998), $\gamma_{t,sj} = (\sigma_t^2 - \sigma_m^2)/\sigma_t^2$, $\sigma^2_{t,sj} = \sigma_m^2\,\gamma_{t,sj}$, and $v^*_j$ is given by the expression

$$v^*_j = \{\exp(\mu)\}^{1-\gamma_{t,sj}} \exp(\sigma^2_{t,sj}/2)$$

As discussed in Reeves et al (1998), when covariates $z$ are included in the regression the mean $\mu$ and variance $\sigma_t^2$ should be those of the conditional distribution of log radon measurements given the values of the covariates for the individual in question. In practice, there was appreciable correlation between only one of the covariates (county of current residence) and the log radon measurements, and so it is enough to estimate $\mu$ and $\sigma_t^2$ separately for the two values of this particular covariate. Thus, for an individual currently living in Devon, $\mu$ and $\sigma_t^2$ were taken as the mean and variance of all the log radon measurements (in whichever county they were measured) for all individuals also currently living in Devon, and similarly for Cornwall. In the present analysis, $\sigma_t^2$ and $\mu$ took values 0.82 and 3.24, respectively, for subjects living in Devon and 1.10 and 4.08, respectively, for subjects living in Cornwall, while $\sigma_m^2$ was estimated externally from a study in which repeat radon measurements had been made (Lomas and Green, 1994). It was found not to differ significantly between dwellings that had the same occupier for both measurements and dwellings with a different occupier, and took value 0.23. This indicates a coefficient of variation on the original scale of 51%.

### *Addresses for which no measurement was available*

For addresses for which no measurement of the radon concentration was available, $X_{sj}$, in equation (1) was estimated using one of the following six methods:

(1) For addresses in Devon or Cornwall for which there was sufficient information to classify the address into one of the six geographical groups described in the section Information on residential radon concentrations the radon concentration was estimated by the geometric mean of all the measurements taken specifically for control subjects in the same geographical group.

(2) For addresses in Devon and Cornwall with insufficient information to assign to a particular geographical group the radon concentration was estimated by the geometric mean of all the measurements taken for control subjects throughout Devon and Cornwall.

(3) For addresses such as houseboats or caravans for which the radon concentration could be assumed to be close to outdoor levels, it was taken to be equal to 4 Bq $m^{-3}$, the typical outdoor concentration in the UK (Wrixon et al, 1988).

(4) For periods at sea, the radon concentration was assumed to be equal to zero (UNSCEAR, 1982).

(5) For addresses in the UK but not in Devon or Cornwall, the radon concentration was assumed to be equal to the estimated geometric mean for the UK, namely 15 Bq $m^{-3}$ (Wrixon et al, 1988, Appendix K).

(6) For addresses outside the UK the radon concentration was assumed to be equal to 30 Bq $m^{-3}$, which was the best available estimate of the world geometric mean concentration (O'Riordan, 1993).

For addresses for which the radon concentration was estimated, $\gamma_{t,sj}$, represents the uncertainty due to measurement error associated with the estimate. For concentrations estimated using method (1) above, from Reeves et al (1998), $\gamma_{t,sj}$, is given by the expression

$$\gamma_{t,sj} = \frac{\sigma^2_{bg}}{\sigma^2_{bg} + (\sigma^2_{wg} + \sigma^2_m)/n_g}$$

where $\sigma^2_{bg}$ is the between geographical group variance of the logarithms of all the radon measurements relating to control subjects, $(\sigma^2_{wg} + \sigma^2_m)$ is the within group variance, which was found to differ between the groups and was therefore estimated separately for each group, $\sigma^2_m$ is the variance of the logarithms of repeat measurements, as for addresses for which a measurement was available, and $n_g$ is the number of measurements in the geographical group g. In fact, $\gamma_{t,sj}$, was found to be very close to unity for all six geographical groups, and it was therefore taken to be equal to unity for all radon concentrations estimated by method (1). For concentrations estimated by methods (2)–(6) above, $\gamma_{t,sj}$, was also assumed to be unity.

For addresses for which the radon concentration was estimated using method (1) above, from Reeves et al (1988), $\sigma^2_{t,sj}$, is given by

$$\sigma^2_{t,sj} = \sigma^2_{bg} + \sigma^2_{wg} - \gamma^2_{t,sj}\,\{\sigma^2_{bg} + (\sigma^2_{wg} + \sigma^2_m)/n_g\}$$

Therefore, when $\gamma^2_{t,sj} = 1$ and $n_g$ is large, $\sigma^2_{t,sj} \approx \sigma^2_{wg}$. For the six geographical groups, the estimated within group variances were 0.52, 0.61, 0.66, 0.92, 0.92 and 1.01, respectively, and, as $\sigma^2_m$ was 0.23, the corresponding estimated values of $\sigma^2_{wg}$ were 0.29, 0.38, 0.43, 0.69, 0.69 and 0.78 respectively. For addresses for which the radon concentration was estimated by method (2), the within group variance based on all the radon measurements taken for control subjects in Devon and Cornwall was 0.98, leading to an estimated $\sigma^2_{wg}$ of 0.75.

For addresses for which the radon concentration was estimated using methods (3) and (4), $\sigma^2_{wg}$ was assumed to be zero. For addresses with radon concentration estimated using method (5) $(\sigma^2_{wg} + \sigma^2_m)$ was estimated from the UK survey (Wrixon et al, 1988) and took value $\{\log(2.17)\}^2 = 0.60$. Therefore, $\sigma^2_{wg}$ was estimated to be 0.37. For addresses estimated using method (6), $\sigma^2_{wg}$ mg was taken to be the estimate from the geographical group with geometric mean closest to the estimated world average concentration, namely 0.43.

© Cancer Research Campaign 1998

### *Radon concentrations adjusted for uncertainties*

The mean time-weighted average radon concentrations adjusted for uncertainties that are given in Tables 8 and 9 are average values of $\Sigma\, w_j v^*_j X_{s(j)}^{\gamma_{L s(j)}}$ for the subjects in question. For each subject, this quantity is the expected value of the true time-weighted average radon concentration given the observed value, conditional on current residence in either Devon or Cornwall.

### *Model fitting*

Maximum likelihood estimates for the parameters in equation (1) were derived by iterative application of the logistic regression command in the Stata statistical package (Statacorp, 1997). For the first step of the iteration, the denominator of the logistic function was assumed to be equal to unity, and in subsequent iterations the numerator was adjusted for the current value of the denominator. In practice, the denominator remained very close to unity for all the models fitted, and therefore confidence intervals for the parameters could be based on the standard errors computed by Stata.

### *Sensitivity analysis*

Additional analyses were carried out to determine the sensitivity of the results to some of the assumptions made above. Firstly, for addresses for which no measurement was available and when $X_{s(j)}$ was estimated using methods (5) or (6) above, the analysis shown in the top line of Table 8 was repeated first doubling and then halving $\sigma^2_{wg}$. The former increased the estimated ERR of 0.12 per 100 Bq m$^{-3}$ to 0.13, while the latter did not change it. Secondly, $\sigma^2_{wg}$ was first increased and then reduced by 20% for all addresses for which there was no measurement available, regardless of the method of estimation of $X_{s(j)}$. The latter increased the estimated ERR from 0.12 to 0.13 per 100 Bq m$^{-3}$, while the former did not change it. Thirdly, the assumed value of $\sigma^2_m$ was first increased and then reduced by 20%. The former increased the estimated ERR from 0.12 to 0.13 per 100 Bq m$^{-3}$, while the latter reduced it from 0.12 to 0.11. Finally, the assumed value of $\sigma^2_m$ was first doubled and then halved. In the former case, the estimate of 0.12 per 100 Bq m$^{-3}$ was increased to 0.16, while in the latter case it was reduced to 0.10.

It was therefore concluded that the results did not depend strongly on the assumptions made in the uncertainty analysis, although, as would be expected, large increases or decreases in $\sigma^2_m$, the variance of the logs of repeat measurements at the same address, increased or decreased the effect of accounting for uncertainties in the analysis.

© Cancer Research Campaign 1998


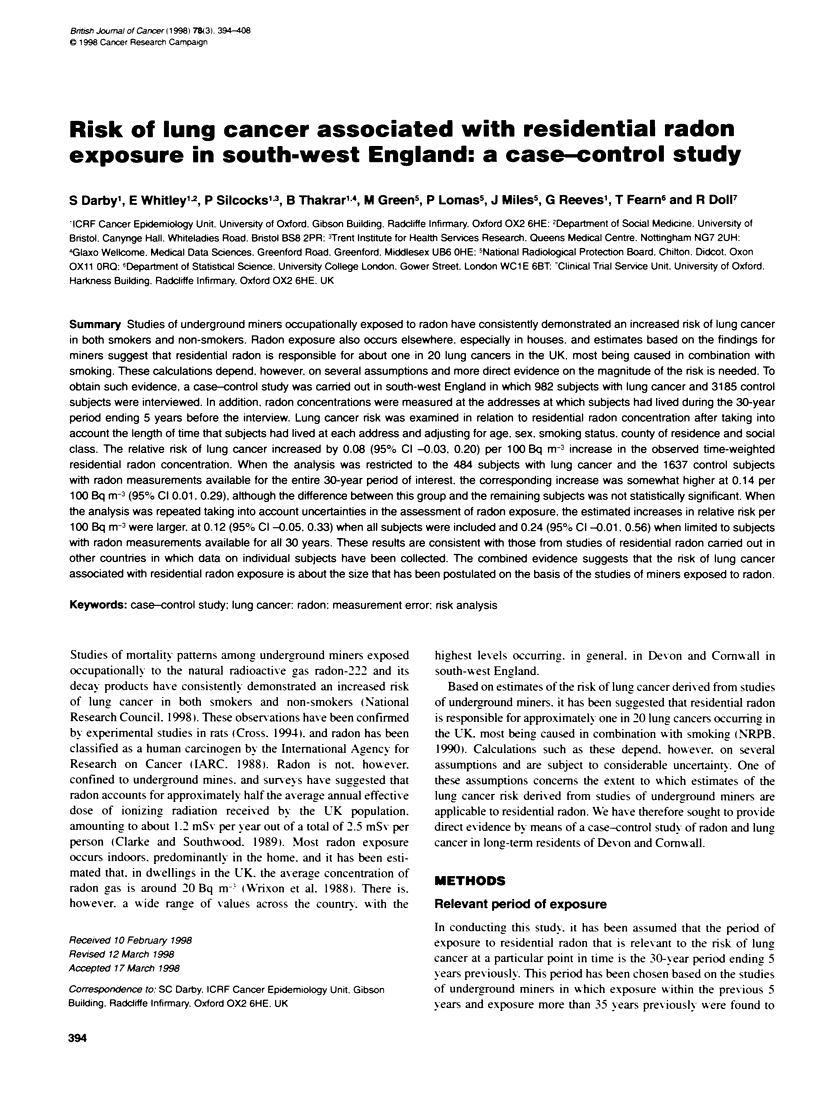

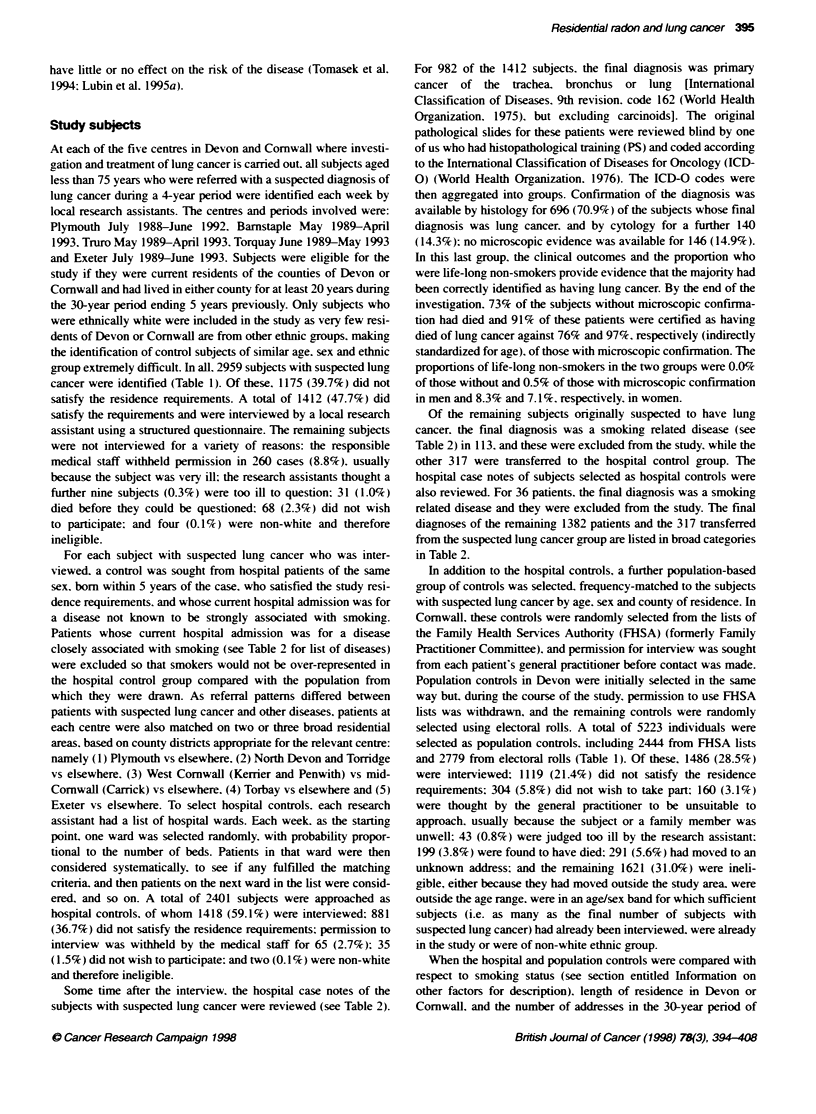

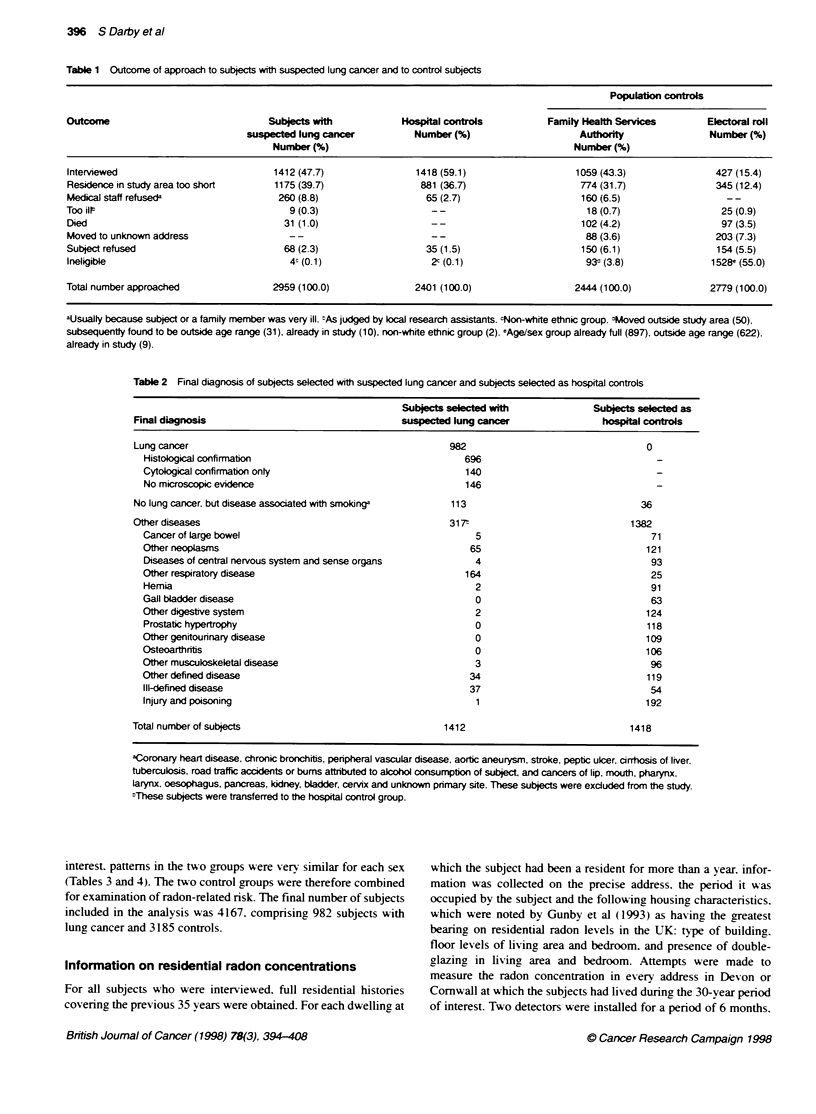

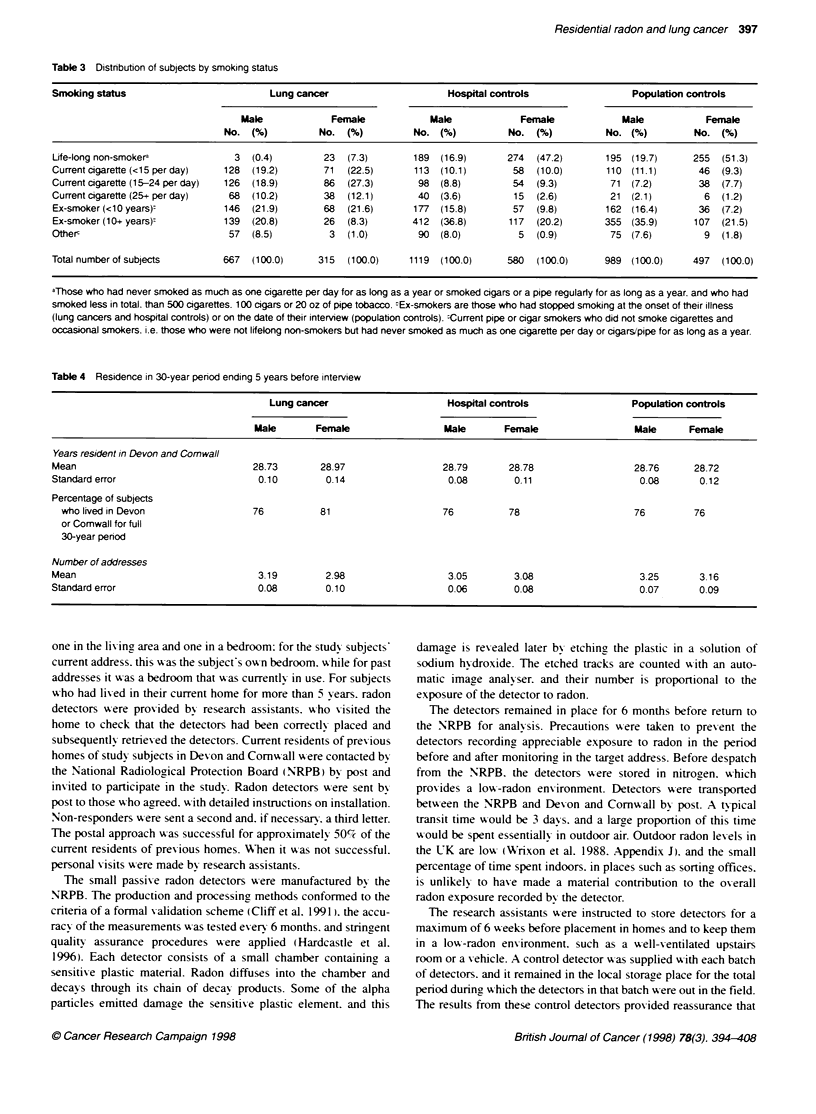

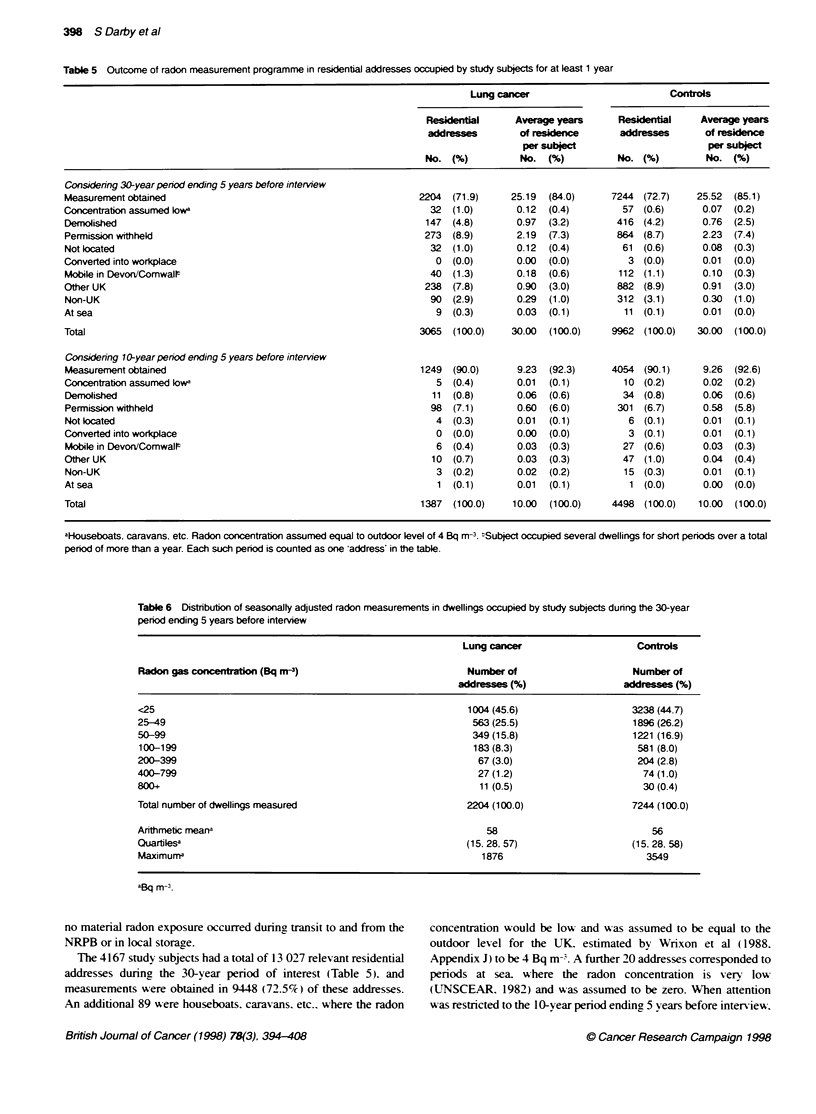

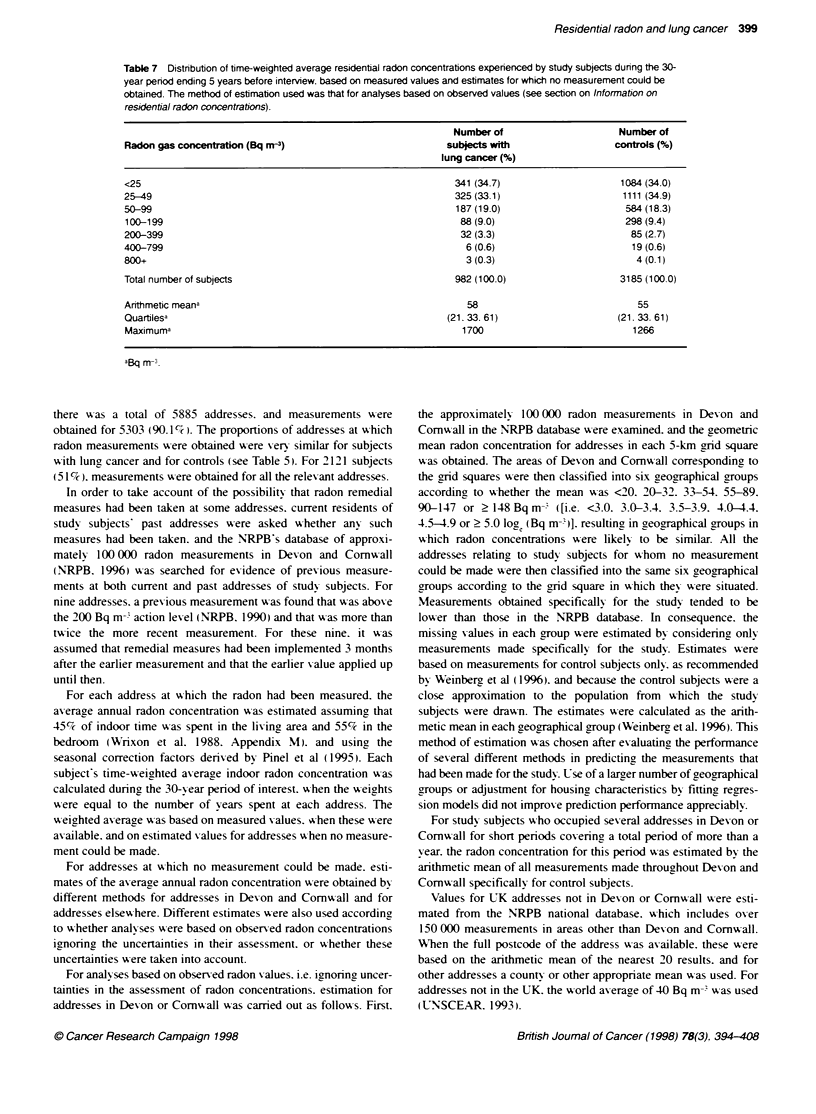

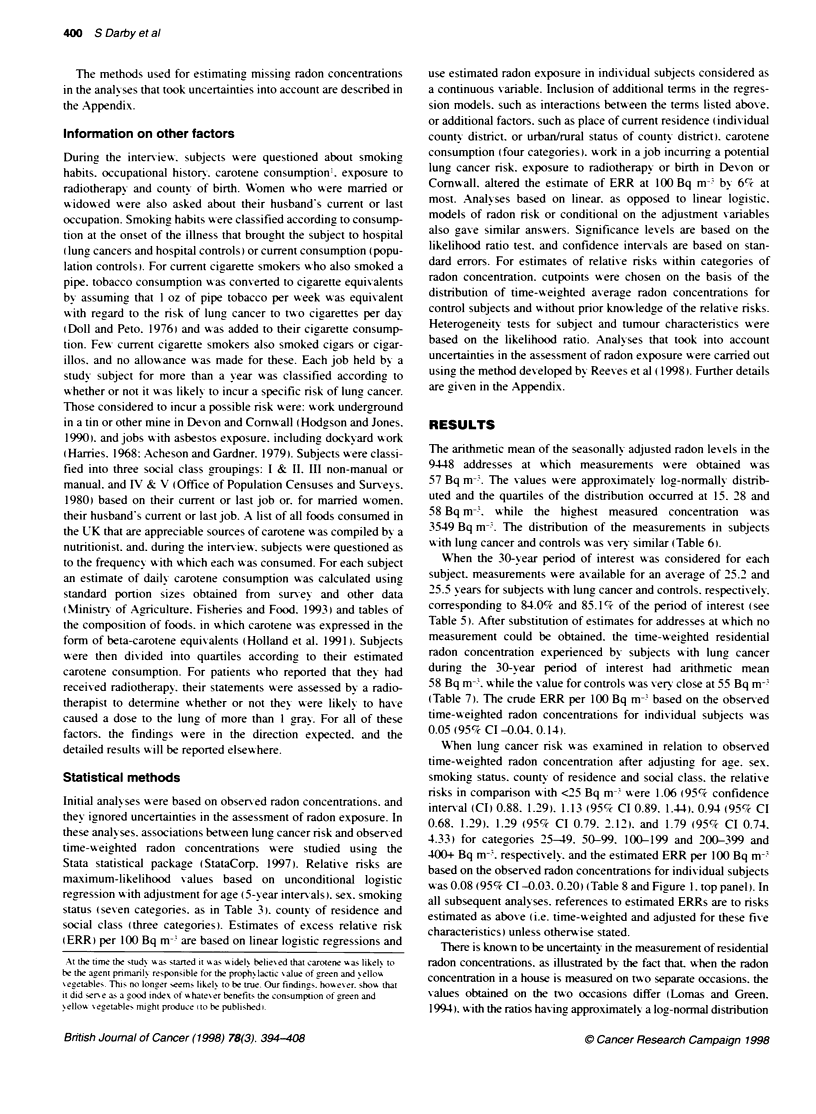

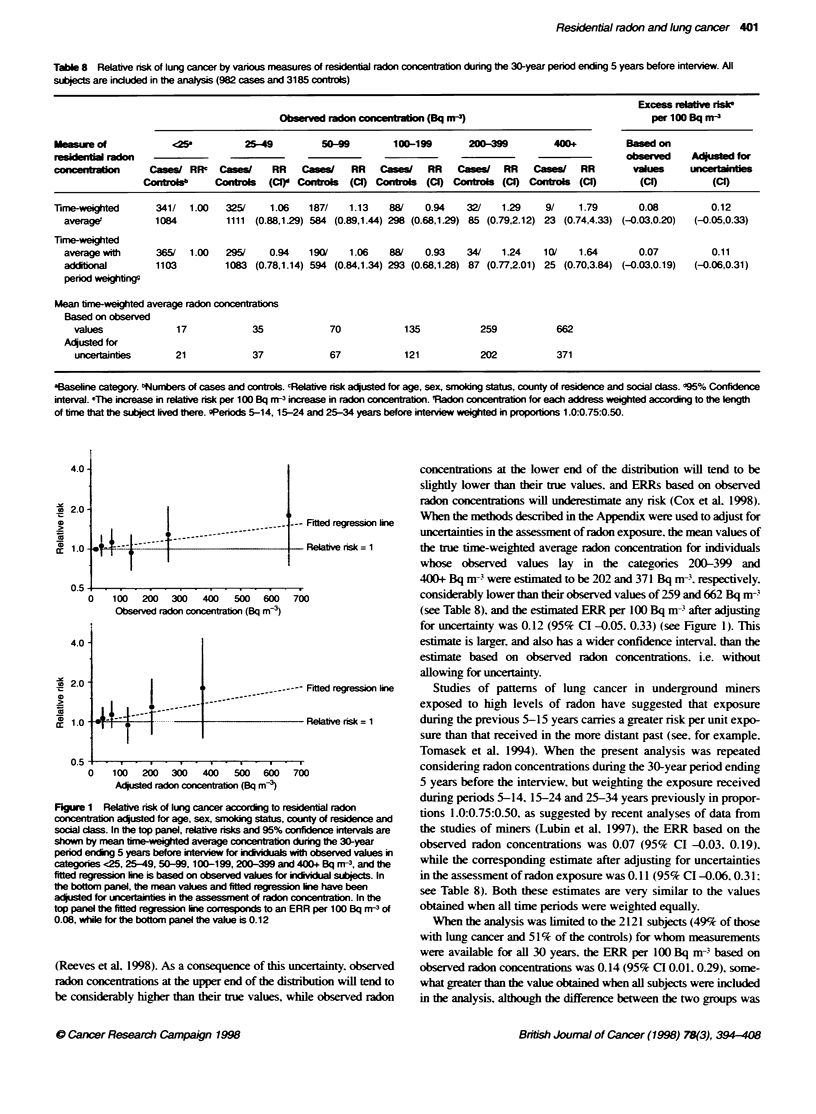

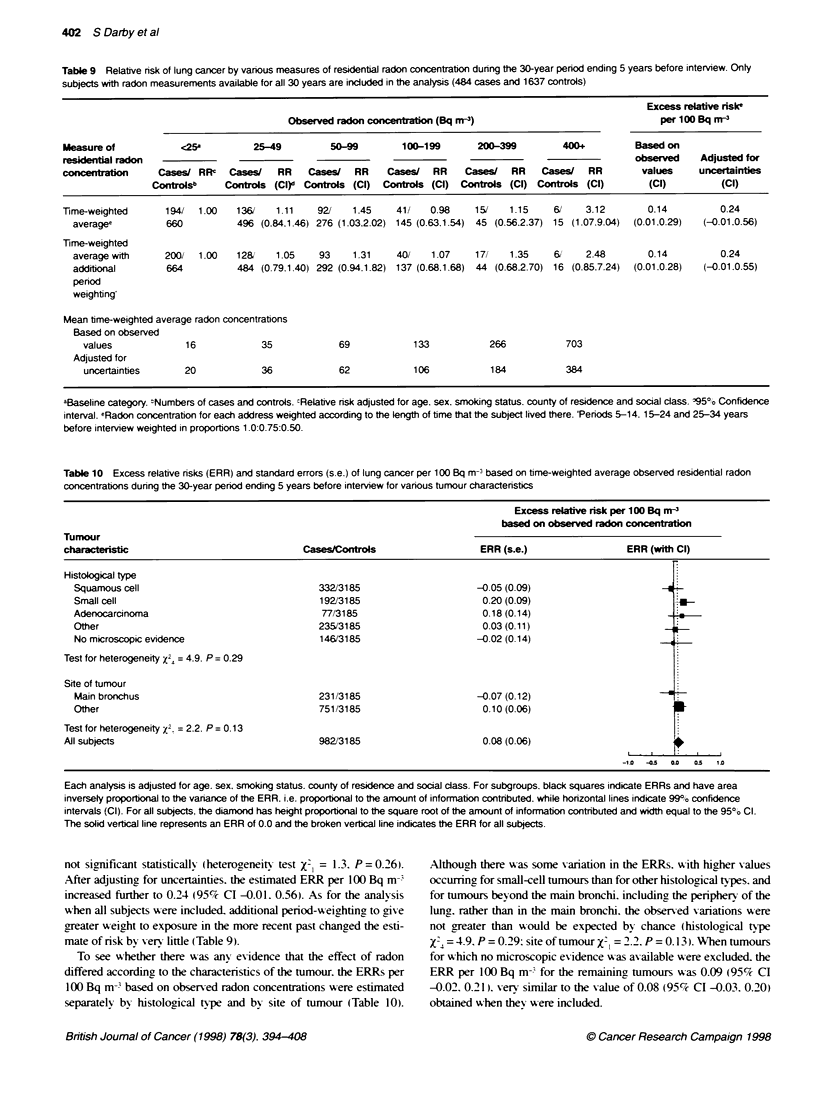

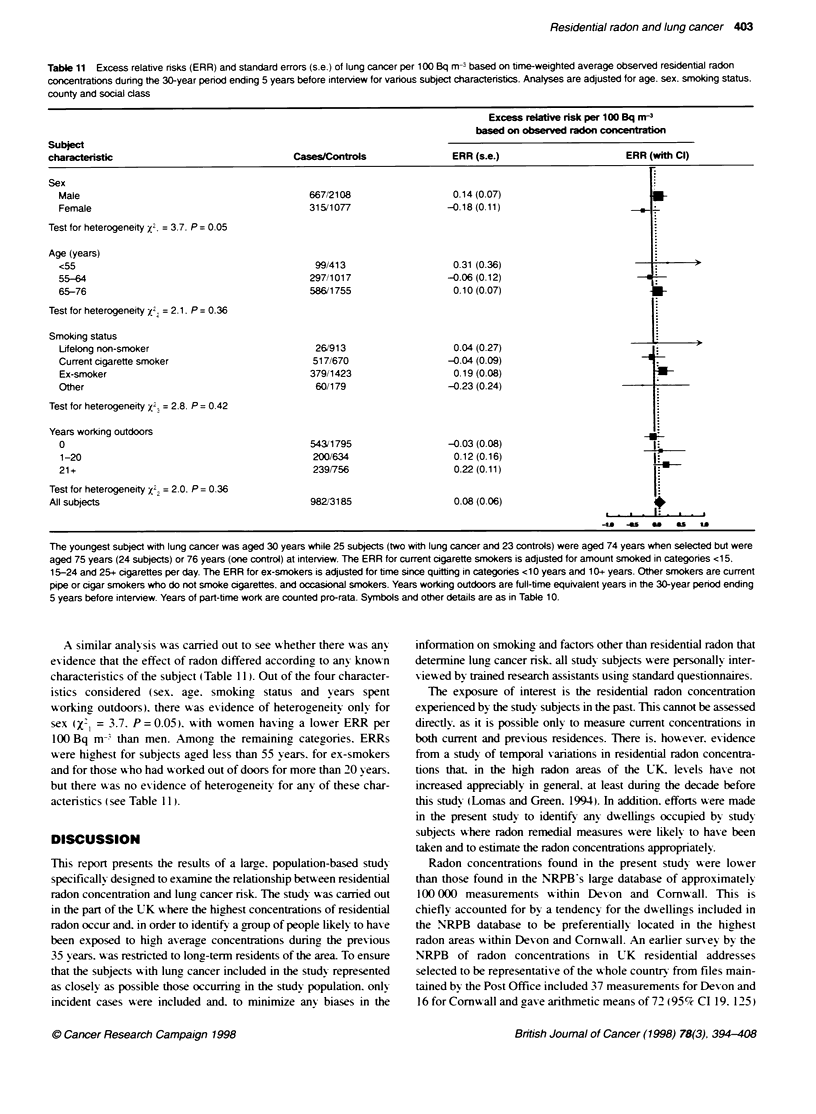

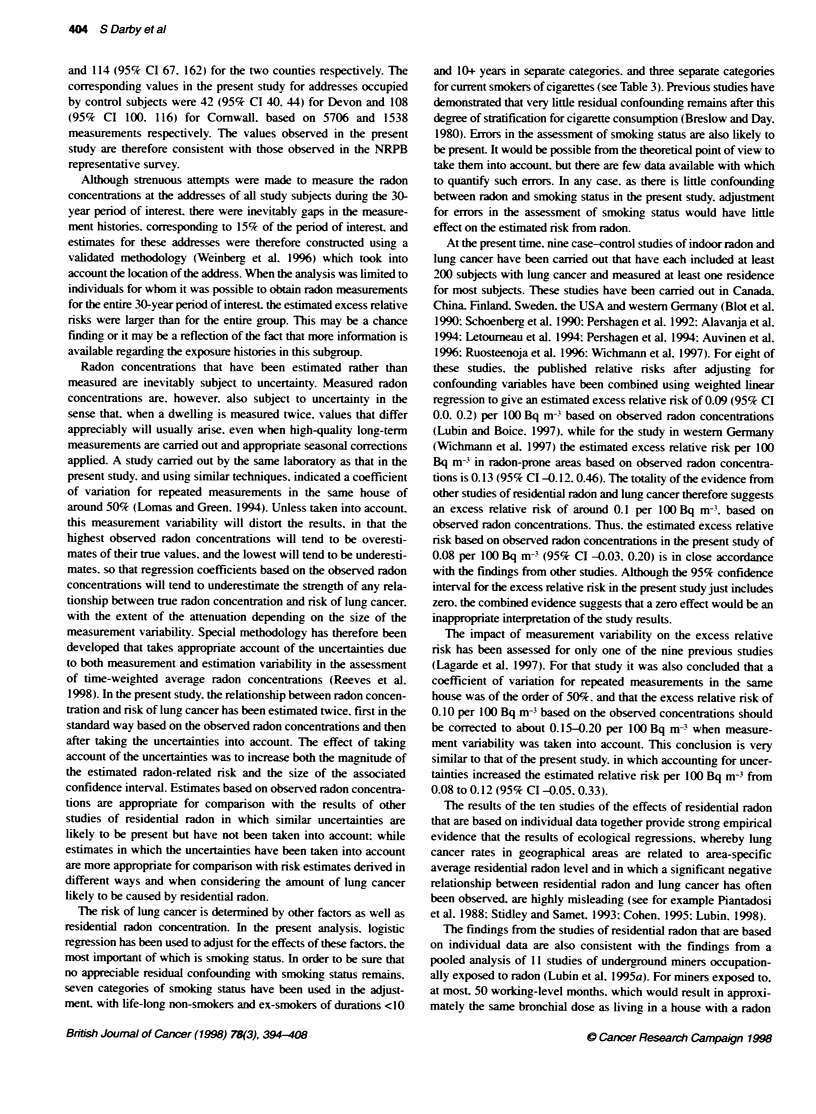

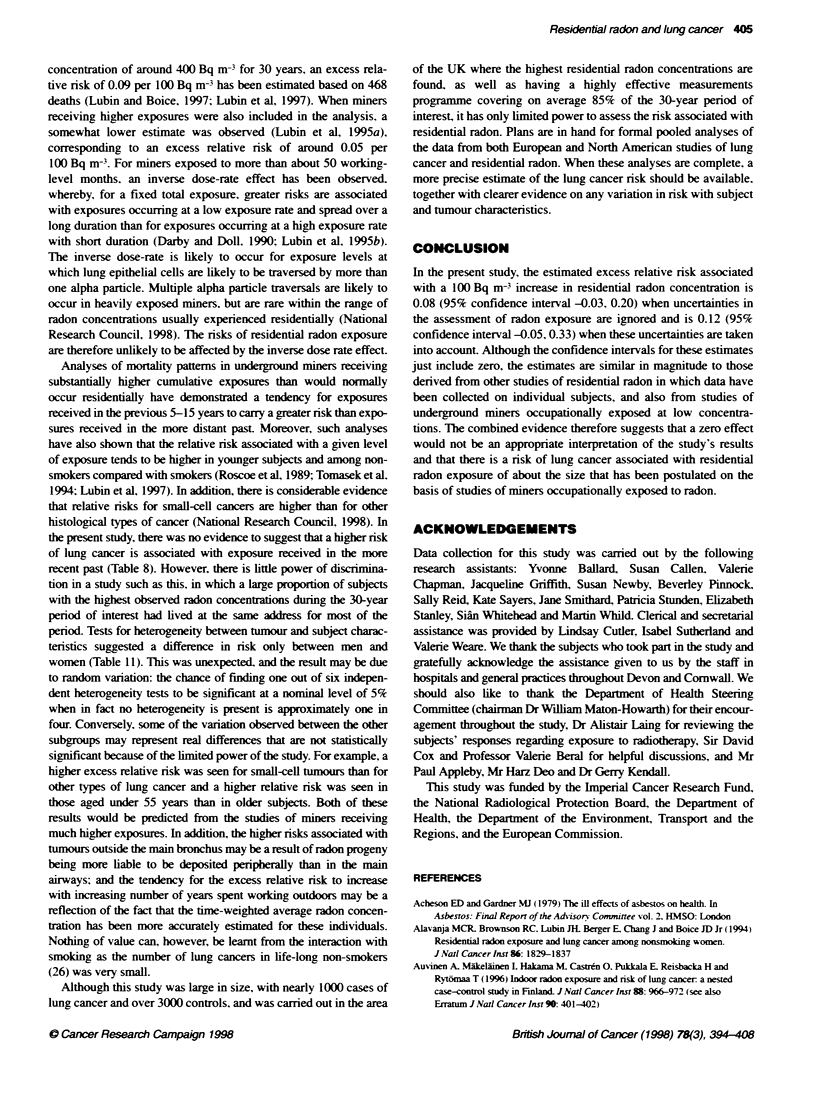

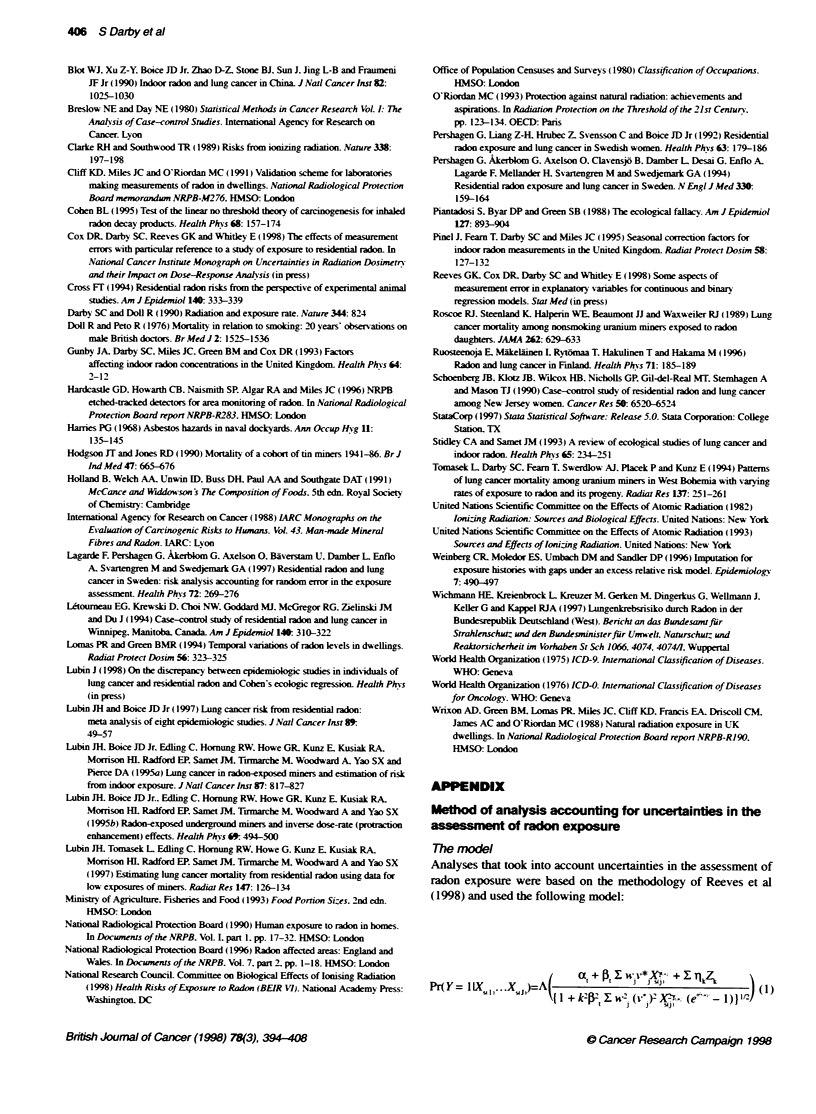

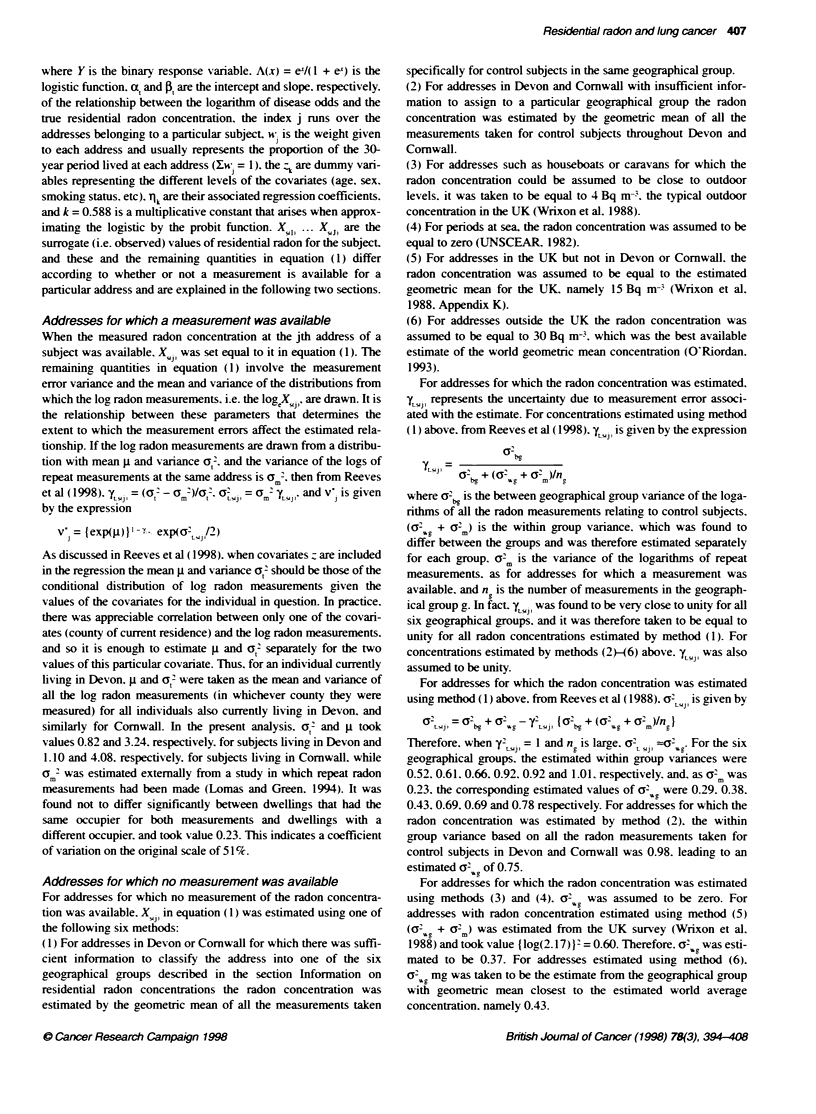

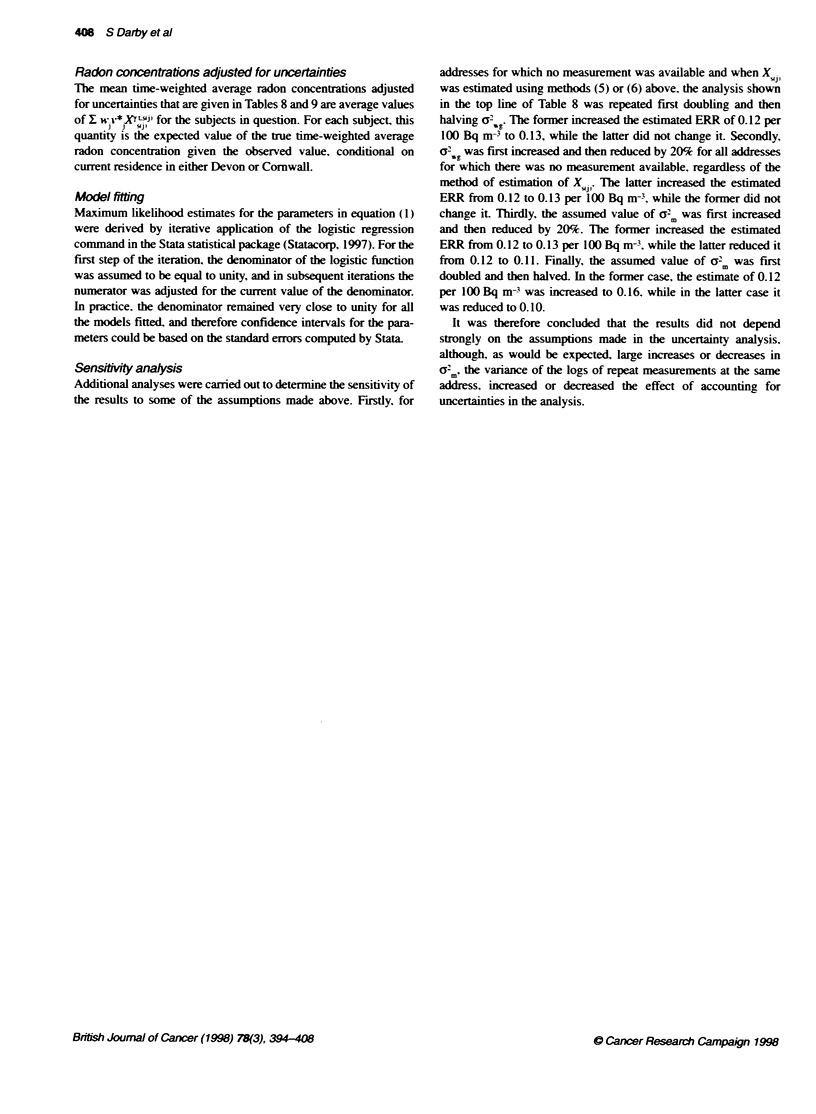


## References

[OCR_01389] Alavanja M. C., Brownson R. C., Lubin J. H., Berger E., Chang J., Boice J. D. (1994). Residential radon exposure and lung cancer among nonsmoking women.. J Natl Cancer Inst.

[OCR_01394] Auvinen A., Mäkeläinen I., Hakama M., Castrén O., Pukkala E., Reisbacka H., Rytömaa T. (1996). Indoor radon exposure and risk of lung cancer: a nested case-control study in Finland.. J Natl Cancer Inst.

[OCR_01402] Blot W. J., Xu Z. Y., Boice J. D., Zhao D. Z., Stone B. J., Sun J., Jing L. B., Fraumeni J. F. (1990). Indoor radon and lung cancer in China.. J Natl Cancer Inst.

[OCR_01412] Clarke R. H., Southwood T. R. (1989). Risks from ionizing radiation.. Nature.

[OCR_01421] Cohen B. L. (1995). Test of the linear-no threshold theory of radiation carcinogenesis for inhaled radon decay products.. Health Phys.

[OCR_01438] Doll R., Peto R. (1976). Mortality in relation to smoking: 20 years' observations on male British doctors.. Br Med J.

[OCR_01442] Gunby J. A., Darby S. C., Miles J. C., Green B. M., Cox D. R. (1993). Factors affecting indoor radon concentrations in the United Kingdom.. Health Phys.

[OCR_01452] Harries P. G. (1968). Asbestos hazards in naval dockyards.. Ann Occup Hyg.

[OCR_01456] Hodgson J. T., Jones R. D. (1990). Mortality of a cohort of tin miners 1941-86.. Br J Ind Med.

[OCR_01470] Lagarde F., Pershagen G., Akerblom G., Axelson O., Bäverstam U., Damber L., Enflo A., Svartengren M., Swedjemark G. A. (1997). Residential radon and lung cancer in Sweden: risk analysis accounting for random error in the exposure assessment.. Health Phys.

[OCR_01498] Lubin J. H., Boice J. D., Edling C., Hornung R. W., Howe G. R., Kunz E., Kusiak R. A., Morrison H. I., Radford E. P., Samet J. M. (1995). Lung cancer in radon-exposed miners and estimation of risk from indoor exposure.. J Natl Cancer Inst.

[OCR_01502] Lubin J. H., Boice J. D., Edling C., Hornung R. W., Howe G., Kunz E., Kusiak R. A., Morrison H. I., Radford E. P., Samet J. M. (1995). Radon-exposed underground miners and inverse dose-rate (protraction enhancement) effects.. Health Phys.

[OCR_01486] Lubin J. H., Boice J. D. (1997). Lung cancer risk from residential radon: meta-analysis of eight epidemiologic studies.. J Natl Cancer Inst.

[OCR_01508] Lubin J. H., Tomásek L., Edling C., Hornung R. W., Howe G., Kunz E., Kusiak R. A., Morrison H. I., Radford E. P., Samet J. M. (1997). Estimating lung cancer mortality from residential radon using data for low exposures of miners.. Radiat Res.

[OCR_01477] Létourneau E. G., Krewski D., Choi N. W., Goddard M. J., McGregor R. G., Zielinski J. M., Du J. (1994). Case-control study of residential radon and lung cancer in Winnipeg, Manitoba, Canada.. Am J Epidemiol.

[OCR_01543] Pershagen G., Akerblom G., Axelson O., Clavensjö B., Damber L., Desai G., Enflo A., Lagarde F., Mellander H., Svartengren M. (1994). Residential radon exposure and lung cancer in Sweden.. N Engl J Med.

[OCR_01540] Pershagen G., Liang Z. H., Hrubec Z., Svensson C., Boice J. D. (1992). Residential radon exposure and lung cancer in Swedish women.. Health Phys.

[OCR_01550] Piantadosi S., Byar D. P., Green S. B. (1988). The ecological fallacy.. Am J Epidemiol.

[OCR_01571] Ruosteenoja E., Mäkeläinen I., Rytömaa T., Hakulinen T., Hakama M. (1996). Radon and lung cancer in Finland.. Health Phys.

[OCR_01582] Stidley C. A., Samet J. M. (1993). A review of ecologic studies of lung cancer and indoor radon.. Health Phys.

[OCR_01588] Tomásek L., Darby S. C., Fearn T., Swerdlow A. J., Placek V., Kunz E. (1994). Patterns of lung cancer mortality among uranium miners in West Bohemia with varying rates of exposure to radon and its progeny.. Radiat Res.

[OCR_01600] Weinberg C. R., Moledor E. S., Umbach D. M., Sandler D. P. (1996). Imputation for exposure histories with gaps, under an excess relative risk model.. Epidemiology.

